# Conceptualizing and treating the polytrauma clinical triad as a complex chronic pain syndrome

**DOI:** 10.3389/fneur.2026.1752206

**Published:** 2026-07-17

**Authors:** Ajay Manhapra, Eamonn Kennedy, David Cifu, Shannon R. Miles, Mary Jo Pugh

**Affiliations:** 1 Hampton VA Medical Center, Hampton, VA, United States; 2 Department of Physical Medicine and Rehabilitation and Psychiatry, Eastern Virginia Medical School, Norfolk, VA, United States; 3 Department of Psychiatry, Yale School of Medicine, New Haven, CT, United States; 4 Informatics, Decision-Enhancement and Analytic Sciences Center, VA Salt Lake City Health Care System, Salt Lake City, UT, United States; 5 Division of Epidemiology, Department of Medicine, Spencer Fox Eccles School of Medicine, University of Utah, Salt Lake City, UT, United States; 6 Department of Physical Medicine and Rehabilitation (PM&R), School of Medicine, Virginia Commonwealth University (VCU), Richmond, VA, United States; 7 Richmond Veterans Affairs (VA) Medical Center, Central Virginia VA Health Care System, Richmond, VA, United States; 8 Mental Health and Behavioral Sciences Services, James A. Haley Veterans’ Hospital, Tampa, FL, United States; 9 Department of Neurosurgery, Brain and Spine, Morsani College of Medicine, University of South Florida, Tampa, FL, United States

**Keywords:** biopsychosocial model of care, chronic pain, interdisciplinary care, polytrauma care, post-traumatic stress disorder, recovery, traumatic barin injury

## Abstract

The polytrauma clinical triad (PCT) - comprised of posttraumatic stress disorder (PTSD), chronic pain syndromes (CPS), and traumatic brain injury (TBI) - is a significant problem among US Service Members and Veterans (SMV). Many SMV treated for PCT improve within 5 years of initial presentation, but a significant fraction show declining health despite extensive care, underscoring the necessity for enhanced management strategies in this population. Since PCT commonly presents as a distressing and debilitating polysymptomatic experience centered around pain, the proposed Functional Recovery Enabling Inter-Disciplinary Evaluation and Management (FREIDEM) model conceptualizes PCT as a complex CPS that emerged and persisted in association with PTSD, TBI, and several other comorbidities. The FREIDEM model integrates several recent clinical advances in conceptualizing and treating complex CPSs and proposes that a complex CPS like PCT represents a state of inadequate or suspended self-healing and recovery due to the cumulative impact of several biopsychosocial impediments (multimorbidity) accrued over a lifetime. The treatment of such complex CPSs is theorized as the advancement of self-recovery with functional interventions, therapeutic mitigation of biopsychosocial impediments to recovery and judicious time-limited use of symptom management including pain management. The FREIDEM model offers a clinical evaluation strategy to identify the biopsychosocial impediments and develop a comprehensive structured treatment plan based on the theoretical model. More research is needed to determine if the FREIDEM model reliably improves outcomes for SMVs with PCT, but it offers a solid framework for treating PCT as a complex chronic pain syndrome and to foster further discussion about new treatment approaches.

## 1 Introduction

The polytrauma clinical triad (PCT) refers to the combination of posttraumatic stress disorder (PTSD), chronic pain syndromes (CPSs), and persistent symptoms associated with traumatic brain injury (TBI), most of which are mild TBI (mTBI). An estimated 13% of US military Veterans enrolled in Veteran Health Administration (VHA) facilities meet PCT diagnostic criteria (1, 2). PCT has also been identified in civilian populations following motor vehicle collisions (3). PCT is associated with adverse outcomes, including substantially higher risk of psychiatric and medical destabilization, suicide, overdose, and overall mortality compared to the general population (1, 2, 4–6). PTSD, post-concussive syndrome following TBI, and chronic pain each represent distinct polysymptomatic disorders but also share overlapping symptoms (4). As a result, the combination of these three PCT components can lead to complex symptom profiles and challenges for clinical management (4). Common comorbidities of PCT include psychiatric disorders, substance use disorder (SUD), medication dependence, polypharmacy, failed treatment, and social stressors (1–4). PCT also interacts with demographic characteristics, with older people with PCT reporting a higher burden of mental illness and chronic conditions compared to the younger PCT population (2).

Many Veterans presenting to VHA facilities with PCT show improvement within 5 years of initial enrollment, but a significant fraction show a worsening clinical picture despite extensive care (2). In practice, this means that a proportion of patients with PCT experience treatment-refractory disabling symptoms with little hope of resolution. Therefore, further work is urgently needed to identify new ways to assist patients with persistent and/or worsening PCT. In response, the VHA and Department of Defense have proposed and implemented an interdisciplinary team-based approach to care for individuals with PCT (4, 7). Although there is guidance regarding the organization and delivery of such interdisciplinary care for PCT, there is no clear guidance regarding the content of care delivered by such interdisciplinary management models when three separate complex component diseases of PCT (CPS, PTSD and mTBI) and several other comorbidities co-exist and create tricky clinical challenges pertaining to patient assessment and treatment (3, 7).

### 1.1 Current models of PCT, their limitations and the need for a new model

Currently, clinicians are encouraged to follow at least seven separate clinical guidelines to manage people with PCT (8). However, there is little guidance on integrating these individual clinical guidelines into a unified interdisciplinary approach for conceptualizing, evaluating and treating people with the complex multimorbid state of PCT (4, 8). To deal with the complexity of care, specialized clinics and programs caring for SMVs with PCT commonly offer multidisciplinary care specific to TBI, and then refer patients to separate multidisciplinary pain and mental health programs treating CPS and PTSD. Case managers coordinate such separate multidisciplinary care using process interventions like templated care plan notes (9). However, such “coordinated” care often fails to meet the needs of the SMV with PCT. In support of this perspective, a qualitative study of providers managing PCT noted “it is challenging to get comorbidities treated by providers who understand cognitive disability that come along with brain injury finding a therapist who can take into consideration the cognitive limitations are hard to find” (10). In addition, several other barriers can compromise the care delivered by existing case managed care models; including loss of information and fall out of patients from care during multiple hand-offs in fragmented care systems, lack of clear clinical lead in organizing and assuring complex specialized care, variability in care, lack or failure of evidence based therapies for comanaging the cooccurring suite of illnesses that accompany TBI, and gaps in knowledge related to long-term rehabilitative care (10–12).

Clinicians have uniformly identified that the ability to provide interdisciplinary whole-person biopsychosocial (BPS) care in-house is a significant facilitator to the delivery of optimal PCT care (13). However, there is no elaboration of the content of such an interdisciplinary BPS model that clinicians can use in practice (14, 15). Thus, there is a need for an interdisciplinary BPS care model for PCT that clinicians can use to deliver most care within specialized PCT clinics, rather than relying on a fragmented care delivery system. This paper develops and describes such an interdisciplinary BPS model of PCT that could be implemented in specialized PCT clinics. By exploring this model, we aim to stimulate further discussions and research avenues that will support patients living with PCT. Although PCT includes the whole spectrum of TBI, this model is specifically applicable for people with mTBI and may not be suitable for people who have experienced a moderate to severe TBI and have severe cognitive compromise. This model has not been empirically tested and should undergo rigorous experimental evaluation before its wide acceptance and integration into practice.

When developing interdisciplinary models of care for PCT, it makes practical sense to anchor the model on one of the component comorbidities. Chronic pain syndrome appears to be the most appropriate anchoring disease due to several reasons, some of which are listed below.

1. Although TBI is the central descriptive feature of PCT that gets the most attention in PCT clinics, chronic pain is often the most disabling symptom that drives perceived care need and delivery. Among people with a history of TBI, individuals with current chronic pain demonstrated notably higher levels of PTSD, anxiety, and depression, and lower levels of sleep quality, community participation, and satisfaction with life compared to people with past pain and no pain (16). Chronic headache, the prominent symptom of TBI, is also a type of CPS (17), and pain is strongly associated with negative outcomes among people with PTSD (18).
2. From a functional perspective, chronic pain has a stronger association with disability than other comorbidities when it is part of multimorbidity conditions. For example, among US adults with PTSD, pain interference has been shown to be a more impactful negative predictor of physical and mental functional outcomes compared to PTSD symptom severity (18). In fact, chronic pain is more debilitating than medical conditions that are commonly associated with disability, like congestive heart failure, renal failure, and stroke (19), and is more debilitating than the core symptoms that define such complex medical illnesses as dyspnea in emphysema or heart failure when they co-occur (20).
3. Compared to TBI and PTSD, CPS has a stronger descriptive classification and mechanistic framework that allows integration of comorbidities (more details in subsequent sections).
4. Chronic pain has a well-developed biopsychosocial framework compared to PTSD or TBI that can be easily adapted to serve as a BPS model for PCT.

Although chronic pain care has a rich tradition of interdisciplinary pain rehabilitation programs (IPRPs) with clear models, the existing models have several limitations that preclude their straightforward adoption in PCT treatment. The existing IPRP models are the successors of the behavioral therapy model for chronic pain proposed and implemented by Fordyce, with a primary focus on pain “management” and functional improvement instead of pain treatment (21). Chronic pain was felt to be interminable and incurable. However, new wave behavioral therapies like pain reprocessing therapy (PRT) have challenged this dogma. A substantial proportion of enrolled patients in a PRT trial reported being pain-free after treatment (22).

The IPRPs typically deliver a combination of psychological interventions, physical therapy, occupational therapy, and pain education using a multi-disciplinary team guided by different theoretical frameworks for each type of treatment (23). For example, psychological interventions may be based on the theories of traditional cognitive behavioral therapy (CBT) or acceptance and commitment therapy (ACT), while pain education is often based on pain neuroscience. However, there is no unifying theoretical framework that integrates these interventions for pain management and those for comorbidities (23).

The biopsychosocial (BPS) model of chronic pain is the anchoring theoretical model that guides much of the IPRPs and most chronic pain care. Although theoretically robust, the translation of the BPS model into a practical treatment model is wanting after 40 years of its existence and acceptance (24). In addition, a clear strategy of integrating comorbidity management into the BPS etiological and treatment framework is also missing. The existing IPRP/BPS models are also anchored on the theoretical concept that the primary utility of pain is to protect the sufferer from harms that are real or imagined. However, persistent pain is better conceptualized as a tool that promotes healing and recovery rather than as a signal of threat (25). The existing models also preceded the modern definitions of chronic pain syndromes as a separate disease (and not a symptom) and the recognition of the dominant role of nociplastic pain in chronic pain syndromes (17, 26).

Thus, any conceptual model or framework of PCT as a CPS should ideally account for the associated multimorbidity, view pain as a part of recovery, and maintain hope for a cure. Such a framework must also integrate treatment for functional recovery, pain, and comorbidities, applying the BPS model and current definitions of chronic pain syndromes and nociplastic pain.

## 2 Conceptualizing PCT as a complex chronic pain syndrome

According to the current description by the International Association for the Study of Pain (IASP) and International Classification of Disease 11th Version (ICD-11), a diagnosis of chronic pain syndrome (CPS) means that the “real” “physical” pain has become a separate problem on its own and is not a symptom of some other physical disease, even if the pain was initially such a symptom (17). CPSs are further subclassified into several chronic PRIMARY pain syndromes without any preceding physical diseases and chronic SECONDARY pain syndromes where pain started as symptom of a physical disease but has since become a problem on its own. It is important to note that this primary and secondary classification of CPSs is a descriptive classification and not to be confused with an etiological or mechanistic classification, meaning that chronic secondary pain syndromes should not be misconstrued as a condition where chronic pain is a symptom of physical diseases at the pain sites. Both primary and secondary CPSs can co-occur with painful physical diseases at the same body site, but they are separate conditions with distinct etiological drivers and treatment approaches.

IASP also clarified that “biological, psychological and social factors contribute to the pain syndrome” in CPS (27). In 2017, the IASP also introduced a new clinical/mechanistic type of pain - nociplastic pain - in addition to nociceptive and neuropathic pain. Nociplastic pain, considered the primary type of pain in CPS, occurs without injury/disease of non-neural tissue or nerves at the pain location (26, 28, 29). This expands the understanding of the etiological drivers of chronic pain to include chronic conditions that are threatening to the whole person- like psychiatric disorders, substance use, chronic medical issues, and psychosocial stressors- even though they are not associated with clear nociceptive or neuropathic triggers. Thus, contrary to common beliefs, CPS is usually not a condition defined by the absence of an identifiable physical cause of pain connected to the pain site, but rather one in which the pain can be driven by nociplastic factors threatening the whole person.

Instead of the traditional conceptualization as a persistent threat signal with protective response, CPS can be seen as a persistent and incomplete healing/recovery response to the adverse impacts of several nociplastic biopsychosocial factors (25, 30). In this view, pain and other symptoms persist together to promote recuperative debility behaviors necessary for healing and subsides once sufficient biopsychosocial recovery is achieved.

Integrating the above concepts, we propose the *Functional Recovery Enabling Inter-Disciplinary Evaluation and Management* (FREIDEM) Model for conceptualizing, evaluating, and treating patients with complex CPSs like PCT. The FREIDEM model conceptualizes PCT as a complex CPS that evolved in association with TBI and physical and/or psychological trauma that has become a separate problem on its own and is not a symptom of past or current physical injuries or diseases. The FREIDEM model further conceptualizes that the lived experience of people with CPSs like PCT represents a behavioral state of recuperative debility sustained by pain and several other symptoms and experiences in response to the cumulative impact of several nociplastic BPS factors accrued over a lifetime. This painful polysymptomatic recuperative debility is likely a part of a complex innate healing response that promotes further recovery in such situations with incomplete recovery and is unlikely to be a passive signal of persistent injury/disease. Pain and other symptoms will likely resolve with full biopsychosocial recovery. So, the FREIDEM model proposes that the etiological evaluation of people with complex CPSs like PCT should focus on identifying nociplastic BPS factors driving the recuperative debility, rather than simply excluding potentially painful physical diseases and injuries (red flags). Effective treatment of complex CPSs like PCT should prioritize rehabilitative interventions and reducing biopsychosocial stressors to support recovery, rather than simply managing pain and symptoms.

**Figure 1:**
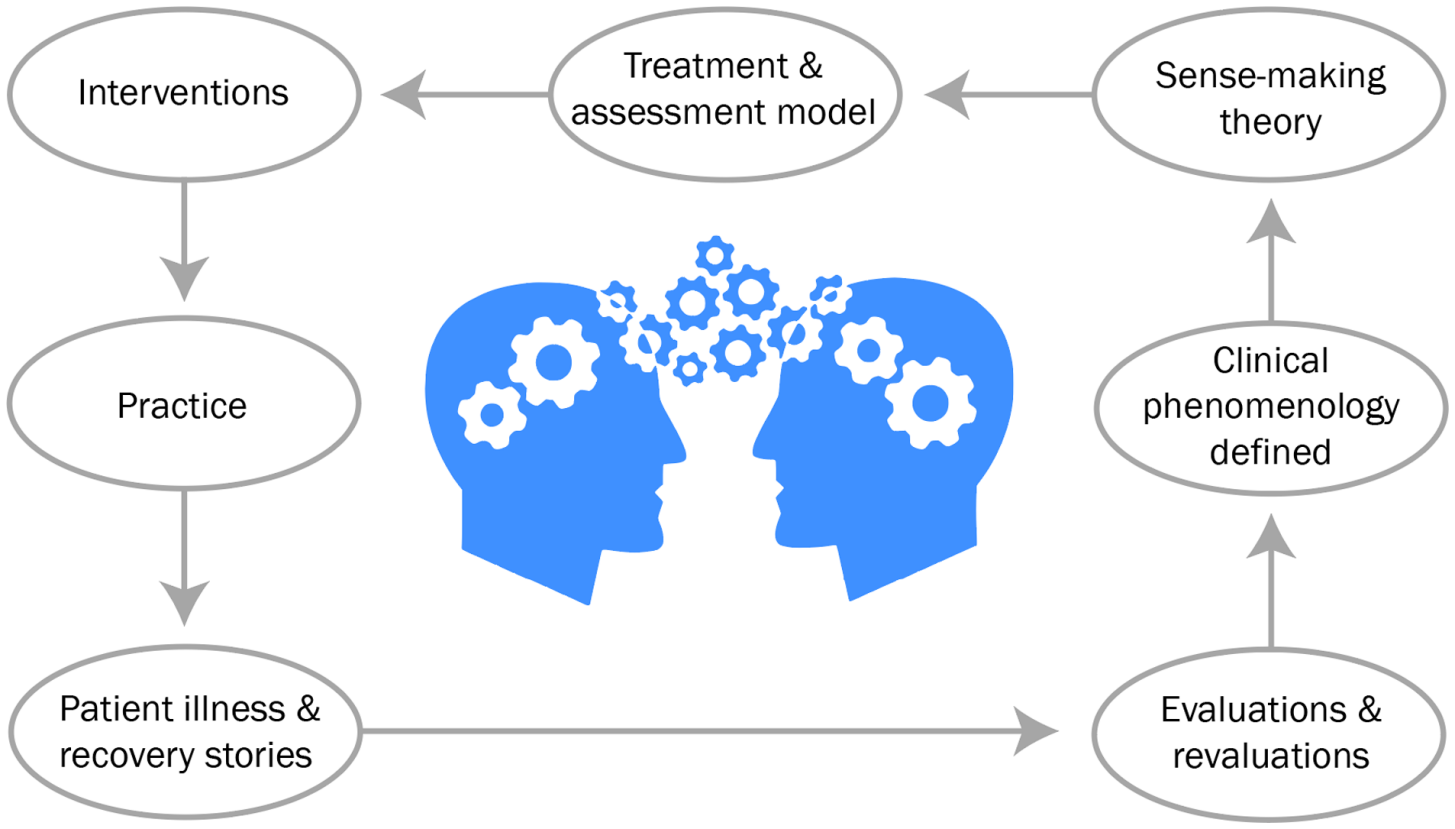
Overview of the functional recovery enabling inter-disciplinary evaluation and management (FREIDEM) model for managing people with chronic pain.

Although the FREIDEM model differs from earlier BPS frameworks, it was developed as an enhancement of these models, motivated by advancements in clinical practice, expanding knowledge, and a more comprehensive understanding of the lived experiences of CPS patients rather than as a completely new theory. This evolution entailed multiple cycles of re-examining patient experiences to gain better insights into the clinical phenomenology of CPS and recovery, applying current knowledge to interpret these insights, and integrating the findings back into clinical practice over 10 years (see Figure 1). The model development was based on the Theory Creation Model proposed by Borsboom et al. (31). In the subsequent sections (Sections 3 and 4), we develop the FREIDEM model following the four sequential steps described by TCM:

Step 1. We describe clinical phenomenology of CPS observed in the clinics (subsection 3.1.),
Step 2. We validate the clinical phenomenology with supporting evidence (subsection 3.2.),
Step 3. We develop an explanatory theory for the clinical phenomenology (subsection 3.3.), and finally.
Step 4. We develop a model that translate the explanatory theory into clinical practice (Section 4).

## 3 Theoretical framework- FREIDEM model

### 3.1 Clinical phenomenology of chronic pain syndromes

The clinical presentation of people with CPSs like PCT is complex, involving several shared symptoms and experiences pertaining to chronic pain, PTSD, long-term sequelae of TBI, several other comorbidities, and psychosocial factors that accompany PCT.

#### 3.1.1 Patient reported pain as a polysymptomatic experience

**Figure 2:**
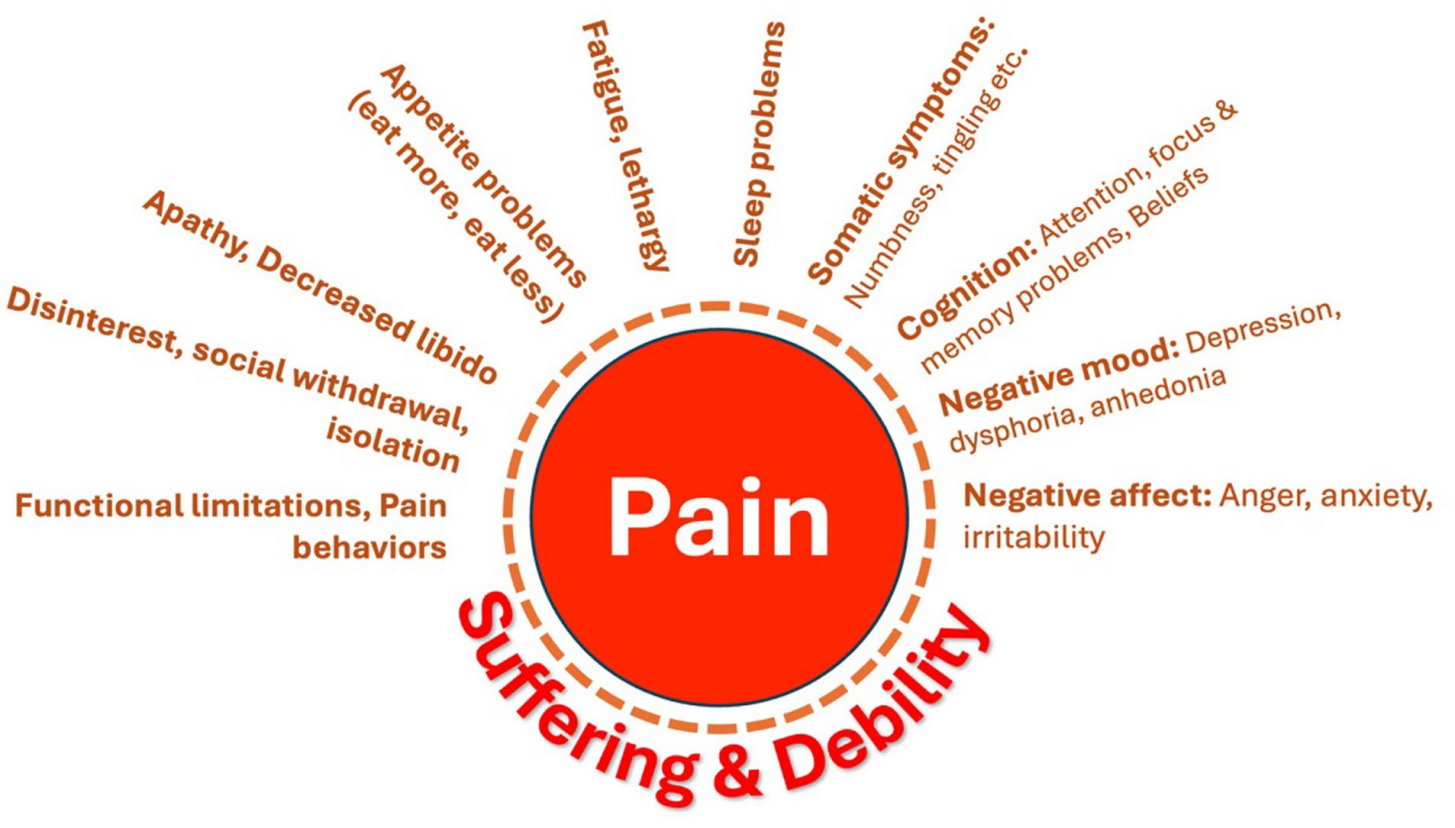
The pain megillah reflects the complex, debilitating, and painful polysymptomatic experience verbalized as “pain” by people seeking care for PCT.

Although the diagnostic and descriptive definitions of CPS are anchored on the singular experience of pain intensity, the lived experience of people who seek care for CPS is more complex and is dominated by a “whirlwind” of several other non-pain symptoms and experiences co-occurring with pain (25, 32, 33). Non-pain symptoms that may contribute to the experience of pain include negative affect and mood, deficits in memory and focus, sleep and appetite problems, somatic symptoms, lethargy, low energy, fatigue, social withdrawal, comorbidities, and functional alterations, all of which may be elevated among people with PCT (Figure 2). Pain and non-pain symptoms commonly develop, persist, and dissipate concurrently rather than as separate, sequential experiences. Speaking metaphorically, pain does not start on Monday, suffering on Tuesday, and debility on Wednesday. Instead, all of them start together on Monday and persists through Wednesday.

The term “pain” usually refers to a singular physical sensation. To draw a distinction, we use the term the pain “megillah” (a Yiddish expression meaning “the whole story” or “everything involved”) as a more comprehensive term to capture the more complex polysymptomatic experience of patients seeking care for painful conditions (Figure 2). Consistent with this complexity notion, palliative care field has long acknowledged the complexity of the polysymptomatic ‘pain’ experience at the end of life and elegantly characterized it as “total pain” (34). For acute conditions, the pain megillah typically resolves with the resolution of pain-related medical illness. In contrast, patients with chronic pain conditions such as PCT frequently report the persistence of pain megillah for many years even after the physical healing of initial physical injuries that might have started the pain response. Recognizing pain as a complex and layered phenomenon is vital for creating comprehensive frameworks about pain, as well as for appropriately assessing and treating individuals with PCT, whose symptoms are frequently perceived and handled as separate problems.

#### 3.1.2 Complex chronic pain syndrome as a multimorbidity experience

**Figure 3:**
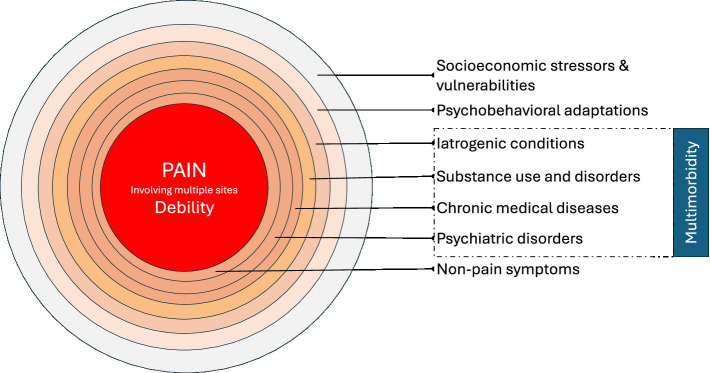
The multimorbid presentation of debilitating chronic pain.

People with severely debilitating chronic pain syndromes like PCT often report several other concurrent problems, including psychiatric disorders, substance use and use disorders, complex medical illnesses, iatrogenic factors like medication dependence, polypharmacy, failed pain management and curative chronic pain treatments, psychological maladaptation, and social stressors. These comorbidities and psychosocial burden and their medical and psychosocial consequences and behavioral expressions often appear wrapped around the central dominant complaints of pain, suffering, and debility (see Figure 3). Thus, debilitating chronic pain is often present as a multimorbidity state where several comorbidities, some of which can be independently debilitating, interact with each other in complex ways and drive the debility associated with pain along with psychosocial stressors (see Figure 3).

### 3.2 Supportive evidence validating the clinical phenomenology of complex chronic pain syndromes like PCT

In this section, we present evidence validating the observed clinical phenomenology we reported in Section 3.1, i.e., the polysymptomatic and multimorbid presentation of people with complex chronic pain syndromes.

#### 3.2.1 Patient reported pain as a polysymptomatic experience

**Figure 4:**
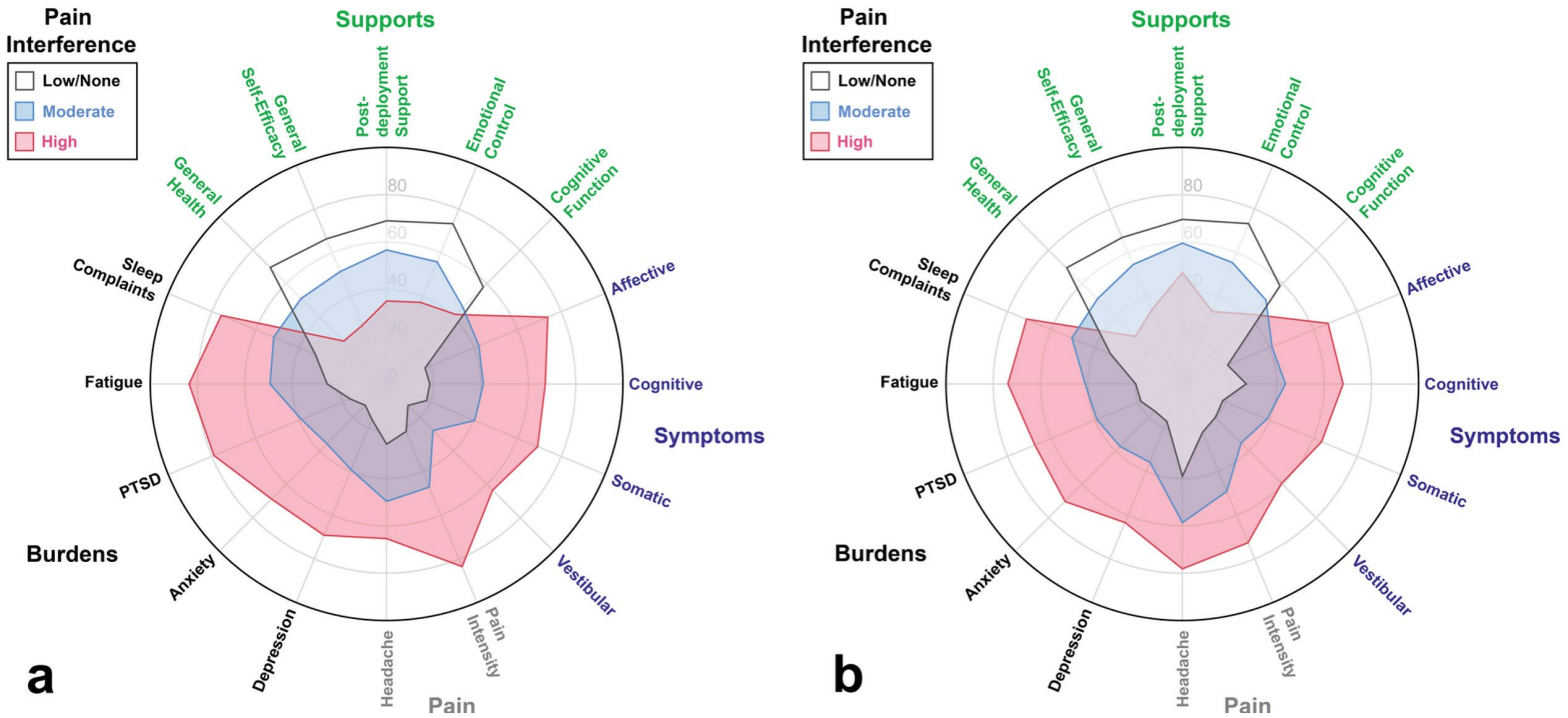
Radial plots visualizing physical and mental health burdens (black), protective supports (green), symptom complaints (blue), and pain measures (gray), broken out by pain interference level (white: low/none, blue: moderate, red: high). Points that are farther out from the center indicate increased values for that variable. Variables were normed to 0–100 to facilitate visualization. Pain intensity level and concurrent non-pain factors among US SMV without **(a)** and with **(b)** history of mTBI showed remarkably similar associations with pain interference, suggesting PCT patients and chronic pain patients experience similar challenges and supports.

Kennedy et al. (35) evaluated factors that may contribute to pain interference conditioned on equivalent pain intensity among combat-exposed US military service members and veterans (SMV) with and without mTBI and with high rates of PCT (36). The study identified that escalation in pain intensity and interference were both associated with escalation of multiple distressing non-pain symptoms, including affective, cognitive, somatic, and vestibular symptoms, fatigue, and sleep complaints and decline in factors that support and promote recovery like sense of general health, resilience, post-deployment social supports, emotional control and regulation, and cognitive function. Machine learning analyses found that pain intensity was only one of several driving factors of pain interference, and non-pain variables like anxiety and fatigue had equal or greater influence on functional impairment compared to pain intensity (36). We conducted a *post hoc* analysis of the same cohort for this paper and found almost identical trends across pain levels, regardless of mTBI status (Figure 4). These data suggest that the clinical phenomenology of PCT resembles other chronic pain syndromes. In a related analysis of adults enrolled in pain clinics, Gilam et al. reported that the severity of “pain-agnostic” features like fatigue, depression, sleep disturbance and interference, anger, anxiety, social isolation, emotional support, and satisfaction were associated with pain severity and debility (33). These findings highlight how non-pain and pain-agnostic factors influence the experience of pain, underscoring the value of a more comprehensive framework like the FREIDEM model for understanding chronic pain syndromes like PCT. Evaluating non-pain and biopsychosocial aspects is crucial for diagnosing and treating PCT effectively with an interdisciplinary perspective.

#### 3.2.2 Complex chronic pain syndromes as multimorbidity experiences

Manhapra and Becker (37) have suggested that a complex chronic pain syndrome like “pain and addiction”, is more than the sum of its parts and is impacted by several additional comorbidities. This is plausible in the case of PCT, too. In this section, we elaborate on observational data supporting this multimorbidity perspective of chronic pain syndromes like PCT.

*Psychiatric disorders*: Prior research has highlighted that the prevalence of psychiatric disorders is notably high among people with chronic pain (19, 38–40). Compared to US adults with low or no pain, individuals with high-impact chronic pain (HICP, i.e., chronic pain associated with functional limitations) report substantially higher prevalence of depression, anxiety, and excessive use of medications (19, 41). Similarly, the prevalence of chronic pain among people with psychiatric disorders was also high. In a study from a national sample, the prevalence of moderate to severe pain interference among US adults with PTSD, a primary component of PCT, was 50%, and it had a stronger association with functional outcomes than the severity of PTSD symptoms (18). Another study reported that the prevalence of debilitating pain (moderate to severe pain interference) was more than twice as high among US adults with psychiatric disorders compared to those without any mental illness (35% vs. 14%) (42). Similar to the expectation with physical disorders, psychiatric diagnosis remission was also associated with lower pain (22% vs. 35%) (42).

*Substance use*: Although the data relating pain to substance use disorders (SUD) is less robust than psychiatric disorders, prior studies have shown that SUD has a complex interaction with chronic pain, whereby current and remitted SUD are both associated with a higher prevalence of debilitating chronic pain (37, 43).

*Distressing chronic medical conditions*: Recent literature has emphasized strong associations of chronic pain with illnesses not commonly considered as painful (21–23). In a recent umbrella review of medical comorbidity and chronic pain, Viderman et al. (20) found that multiple conditions that were not typically associated with chronic pain by clinicians were, in fact, associated with a notably high prevalence of chronic pain. For example, nearly 50% of people with end-stage renal disease, 60% of people with Parkinson’s disease, 60% of patients with chronic obstructive pulmonary disease (COPD), and up to 85% of patients with congestive heart failure (CHF) report significant persistent pain (20). The functional impact of pain in some of these chronic diseases is even higher than the cardinal symptoms of these diseases/conditions (e.g., dyspnea in COPD and CHF) (20). In comparison, just 20%–30% of patients with radiographic osteoarthritis of lower extremity joints report pain (44–47).

*Multimorbidity*: Large epidemiological studies have noted a high prevalence of multimorbidity among US adults who report high-impact chronic pain, and the disability increases as the number of comorbidities rises (19, 41). A higher burden of multimorbidity is also associated with serious adverse outcomes like suicides among people with chronic pain (48, 49).

*Iatrogenic factors*: Unsuccessful treatment of the presumed physical reasons of chronic pain is often associated with worsening chronic pain, for example, failed back surgery syndrome (50, 51). Medication dependence arising from long-term pain management also increases the risk for chronic pain (52, 53). A common strategy for treating chronic pain is rational polypharmacy targeting different pain mechanisms and other symptoms associated with pain (54, 55). However, chronic pain often remains intractable or worsens despite such “rational” polypharmacy (36, 56–58). The high prevalence of comorbidities further intensifies polypharmacy (19, 36, 37, 56–61) and can lead to increased fatigue, lethargy, somnolence, drowsiness, insomnia, and other related complications (56, 57, 59, 60, 62). Thus, although done with good intentions, polypharmacy is associated with worsening pain and the emergence of new symptoms in complex CPSs like PCT (37, 58).

*Psychosocial factors*: Decades of research have established that psychological adaptations like fear avoidance behaviors, catastrophizing, lower self-efficacy, loss of control, and negative emotions, beliefs, and thoughts have a significant association with pain interference (63). Research has also shown that social stressors can occur concurrently with chronic pain in ways that can drive increased distress and debility (64).

In summary, several lines of evidence support the view that the observed phenomenology of complex chronic pain syndromes like PCT is not confined to the singular experience of pain but is a whirlwind of concurrent symptoms, i.e., the pain megillah, in close association with multimorbidity.

### 3.3 Explanatory theory of the clinical phenomenology of chronic pain syndromes like PCT

In Section 2, we introduced the FREIDEM model, an explanatory framework for chronic pain syndromes (CPS) such as PCT. This model links the clinically observed polysymptomatic presentation (“pain megillah”) and its association with multimorbidity to contemporary understandings of chronic pain and recovery processes. The FREIDEM model conceptualizes CPS, including PCT, as a condition of incomplete healing and recovery resulting from the cumulative effects of multiple nociplastic biopsychosocial (BPS) factors acquired throughout life. Treatment strategies for CPS within this model are oriented towards rehabilitative interventions that enhance or expedite the body’s intrinsic healing and recovery mechanisms, as well as approaches that address BPS barriers to recovery—rather than relying on long-term pain and symptom management.

The following sections address key conceptual domains essential for developing the FREIDEM explanatory model: (a) pain and recovery; (b) the pain megillah as a therapeutic instrument; (c) recovery based functional definitions of acute and chronic pain: (d) nociplastic pain and the diagnostic criteria for chronic pain syndrome; (e) the biopsychosocial framework; and (f) the impact of repetitive relief efforts on pain and recovery. The concluding section then synthesizes these topics within the context of the FREIDEM model.

#### 3.3.1 Pain megillah and illness recovery

When developing a model for chronic pain syndromes like PCT, it’s important to first consider the purpose of pain. One belief is that pain serves to warn and protect the individual from threats. However, as early as 1979, Patrick Wall dismissed the threat warning and protection agent functions of pain, stating, “pain is taken not as a simple sensory experience signaling the existence of damaged tissue … Pain is a poor protector against injury since it occurs far too late in the case of sudden injury or of very slow damage.” Instead, he proposed, “pain signals the existence of a body state where recovery and recuperation should be initiated” (65). It is now well established that in painful illness states such as PCT, pain is a call to action to initiate behaviors that favor healing, recovery, and survival after threats. Pain acts as an instrument to escalate, calibrate, and de-escalate healing behaviors essential for recovery (66).

The best scientific definition of healing originates from the field of hospice care. Summarizing the decades of experience from the hospice world, Namisango et al. (67) state that “healing occurs when patients experience positive social and psychological changes in their lives irrespective of the disease-related outcome.” They further elaborate that “…*healing is multi-dimensional, subjectively experienced differently by individuals, and spans physical, social, spiritual, and psychological domains of well-being”* (67). Distress and pain abate among hospice patients who experience healing, and they often experience joy. Healing should ideally be conceptualized as finding a “new normal” functional life that aligns with the individual’s values, with a body that has learned from suffering a threat, rather than reverting to the “old normal” with the old body. Healing can be defined as positive changes across many domains of functional life after facing a threat, while recovery is the process of achieving healing.

#### 3.3.2 The pain megillah as an instrument for healing and survival

When facing serious physical or emotional threats, an individual’s physiological systems must initiate automatic behavioral and biological adaptations to facilitate healing and ensure survival (66, 68). To foster healing and survival, the recovering body reallocates resources from routine health functions to those essential for immediate healing and survival (66, 68). For example, when faced with a threat like infection, the body prioritizes physical rest, redirecting limited energy toward processes such as mobilizing the immune response, which is essential for fighting the infection and ensuring survival (66, 68).

To quickly and automatically reallocate resources in response to the shifting needs of the state of healing, the body constantly balances the innate motivational drive to function with the concurrent drive to rest and heal (66). Thus, the body’s functional behavior during recovery reflects the sum of the degrees of the two key opposing motivational drives to function and to rest. This motivational dynamic appears to manifest clinically as a preferential debility, in which essential physical activities with high motivational value are allowed despite the potential to cause more pain and distress, while non-essential, low-motivational activities are suppressed, even though they are less painful and distressing (66, 68, 69). For example, following an injury or infection, the individual feels fatigued, painful, and distressed, and rests in bed most of the time. They are unable to perform mundane, non-essential, low-value activities like cleaning the house or doing chores, but their bodies allow essential activities like visiting a physician or taking care of their young children, despite the substantial risk of increased pain and distress. Such preferential debility is an adaptive response in acute sickness but can be unhelpful if it persists as a chronic response (69). We often observe that some people with chronic pain can do 8 hours of manual labor at work to make a living (essential activity that can potentially cause a lot of pain) but cannot get off the couch and do simple tasks when they return home after work (non-essential activities that are potentially less painful). Chronic pain literature has described such preferential disability driven by motivational dynamics related to competing goal pursuits where patients become less sensitive to pain when pursuing valued goals (70, 71).

Motivational experiences like pain, depression, anxiety, fatigue, lethargy, depression, sleep alterations, and cognitive changes are the motivational instruments the body uses to automatically enforce this *recuperative debility* and the whole recovery response (66, 68–71). Thus, the polysymptomatic experience that patients describe as “pain” (pain megillah) is an instrument that the body can leverage to calibrate healing and survival behaviors following exposure to threats. As seen in sickness behaviors, the pain megillah serves to balance the body’s drive to rest and heal (recuperate) against its drive to pursue activity and life goals needed for survival (30, 66, 68). In summary, pain reflects the body’s efforts to recover from an injury or threat rather than the mere existence of such injury or threat.

The body’s recovery response to threats can be expected to resolve and will likely do so only after the body has achieved the maximum possible functional stability compatible with survival (30, 68). Thus, as commonly misconstrued, the therapeutic reduction of pain is not required for recovery after a threat. Instead, pain is a necessary component of recovery, and lasting resolution of pain is possible only after sufficient recovery. For example, people experience debility and distress with the pain megillah following a knee surgery. Many cannot go for a leisurely walk, a non-essential activity with low intrinsic motivational value, “because it hurts too much”. However, they can commonly perform essential activities with high intrinsic value, such as sitting up for meals, going to the bathroom, or participating in physical therapy, although these activities can cause substantially more pain than non-essential activities. During the post-operative period, patients slowly advance their non-essential activities with pain, and the pain resolves with escalation of activities. People who insist on the resolution of pain before resuming activities tend to have more pain, stay relatively immobilized, and ultimately do not experience recovery.

#### 3.3.3 Recovery-based functional definitions of acute and chronic pain

The above concepts of pain and recovery lead to a functional definition of both acute and chronic pain conditions that is pertinent to recovery-focused treatments. Acute pain indicates the initiation of a multidimensional recovery response to threats, the pain megillah, which self-terminates after the achievement of sufficient healing and recovery supportive of sustaining life. Chronic pain with the long-term persistence of the pain megillah indicates a state of ongoing, interrupted, or stalled innate healing and recovery. It is now obvious that ***lasting*** reduction of pain and other distressing symptoms, the primary goal of all patients suffering chronic pain, is possible only if the person with pain can advance or accelerate functional recovery WITH pain (self-recovery). Mitigating impediments to recovery is also essential to fostering self-recovery. Treatments solely focused on immediate reduction of pain, targeting various pain mechanisms, will not likely lead to recovery by themselves.

#### 3.3.4 Nociplastic pain

A common belief is that pain and other symptoms among people with CPS, like PCT, are the result of physical tissue damage or neural injury from mTBI and other conditions. However, when evaluated, the majority of patients with mTBI have negative imaging findings, and many attribute their pain to other unrelated conditions (72). This apparent contradiction is alleviated by modern conceptualizations of pain types. There is increasing recognition that chronic pain experiences do not easily fit into one of the two widely recognized types of pain: nociceptive pain, related to tissue injury or disease, or neuropathic pain, related to peripheral neural injury or disease. In response, a third category, “nociplastic pain,” was introduced in 2017 by the International Association of the Study of Pain (IASP) (26, 28, 29). Nociplastic pain is defined as pain arising from altered central nervous system mechanisms of pain generation, without clear evidence of injury or disease of tissues or the somatosensory nervous system (26, 28, 29). An important clinical characteristic of nociplastic pain is its strong association with non-pain symptoms like fatigue, depression, anxiety, sleep problems, and cognitive challenges, and its tendency to be regional or widespread in distribution (29) –clinical characteristics that define the lived experience of people with CPSs like PCT. The concept of nociplastic pain was a paradigm shift in chronic pain treatment, but has not been applied to understand chronic pain syndromes like PCT. This shift clarified that the “real” or “physical” pain reported by people with chronic pain syndromes like PCT can often develop in association with “non-physical” conditions without any significant contributions from physical injuries or diseases. So, the causes of such nociplastic pain are not evident on X-rays or MRIs.

Importantly, the pain megillah among people with CPS like PCT often presents and persists for years despite no underlying physical non-nociplastic (nociceptive or neuropathic) drivers. One interpretation of these observations is that *pain and other symptoms persist because the other PCT components continue to trigger pain and associated symptoms through nociplastic mechanisms*. Evidence for this includes strong associations between chronic pain and diseases that lack clear nociceptive or neuropathic sources. For example, PTSD, a core PCT component, has no clear nociceptive or neuropathic mechanism of pain generation, yet has a remarkable association with persistent pain that is not accounted for by nociceptive or neuropathic comorbidities (35, 42). A similar pattern of pain chronification is observed for substance use disorders (37, 43, 73) and other non-pain medical conditions (20) that are commonly associated with PCT. Furthermore, the prevalence of pain associated with non-physical conditions like PTSD and other mental illnesses is often higher than that caused by conditions commonly associated with nociceptive pain, such as osteoarthritis (20, 35, 37, 42, 43, 73, 74). Thus, medical interventions intended to achieve lasting resolution of pain and other symptoms among people with PCT might have to primarily target the PTSD and other underlying nociplastic comorbidities rather than incidental non-nociplastic conditions revealed on radiographs and other investigations.

#### 3.3.5 Biopsychosocial model

The biopsychosocial (BPS) model is a widely accepted framework in chronic pain research and clinical practice, used to explain the pain experience and its complex, proximal causal drivers. So, it is important to situate the recovery model for chronic pain syndromes like PCT (as stated in sections 3.3.3) using biopsychosocial model foundations. The IASP has stated “chronic pain is multifactorial” and “biological, psychological and social factors contribute to the pain syndrome” (27). However, a clinically useful/practical restatement of the BPS model of CPSs, such as PCT, including the context of nociplastic pain, its less recognized proximal drivers, and a recovery model of CPS, is not yet available for clinicians to use in clinical practice. In addition, the available versions of the BPS model in chronic pain mostly consider nociceptive and neuropathic conditions as the biological drivers. This is difficult to reconcile with the fact that most chronic pain conditions are nociplastic (75), and that the strongest association of pain in CPSs is with non-physical factors not related to tissue or neural damage (36). Thus, the biopsychosocial model for PCT may benefit from realignment with explanations of nociplastic chronic pain and recovery model.

When proposing the biopsychosocial model, Engel was clear that it was not a holistic or humanistic model, both of which were considered dogmas, but a *scientific biomedical model for biomedical practice* to replace the prevailing dogma, namely the biomedical model that left no room for psychological or social factors to be considered as etiologies and treatment targets for diseases or the associated illnesses (76). Put simply, the biopsychosocial model states that the illness state is experienced by the whole person, not by the individual component organ systems affected by the disease pathology. So, while molecular-level pathologies that affect the brain and other parts of the physical body may contribute to the symptoms of complex medical conditions like CPS, the biopsychosocial model asserts that the illness states experienced by the whole person is much more complex and that a myriad of factors that affect the whole person, and not just the diseased organ, drives the experiences of illness and the relief and recovery from it. Engel categorized such factors into three groups: biological, psychological, and social- creating the whole-person biopsychosocial model of illness and its treatment (76). As per the BPS model, biological diseases can be generated by psychological and social distress and vice versa. Hence, treatment of people with illnesses should target all three elements of BPS model and not just the biological diseases. When evaluating people with illness, providers and patients should recognize that physical illnesses can be caused by psychological and social factors without major structural biological changes. A concise list and categories of biopsychosocial risk factors that contribute to the evolution and experience of chronic pain illness state are provided in Box 1.

**Box 1. Biopsychosocial risk factors contributing to the illness state associated with chronic pain diagnoses.**

**Biological factors**

- Biological vulnerabilities

*Genetic factors, gender, socioeconomic and educational status, etc.*

Persistently distressing medical diseases*

*Psychiatric disorders, substance use and use disorders, distressing chronic medical illnesses, and iatrogenic conditions*

**Psychological factors (Nociplastic)**

*Fear avoidance behaviors, low pain self-efficacy, pain catastrophizing, negative beliefs etc.*

**Social factors (Nociplastic)**

*Life, employment and relationship stress, loss of self-identity, employment, relationships and social roles, lack of resources and healthcare access, etc.*

*A more elaborate list of persistently distressing medical illnesses for clinical practice:

***Psychiatric disorders:*** Include trauma, post-traumatic stress disorder, depression, anxiety and severe mental illnesses.

***Substance Use:*** Include regular medical and non-medical substance use for relief and euphoria, active and remitted substance use disorders are included.

***Distressing chronic medical illnesses:*** Include Congestive heart failure, chronic obstructive pulmonary disease, cancers, post-covid illness, critical illness survivorship, post-bariatric surgery state, traumatic brain injury and concussion, etc.

***Iatrogenic conditions:*** Include Medication dependence (Opioids, sedatives, benzodiazepines, gabapentinoids, muscle relaxants, stimulants, and other psychoactive medications), polypharmacy especially with addictive medications, overuse and failure of pain management (pharmacological, non-pharmacological and procedural), and failed curative treatment of presumed physical reasons for chronic pain (failed back surgery syndrome, failed arthritis and chronic inflammatory disease treatments, etc.).

In this rendering of the BPS model, we propose that individual biopsychosocial factors are not singular causes but risk factors that interact in complex ways to generate the illness experience (the whole pain megillah) associated with complex illnesses like PCT. To align the model better with clinical practice in chronic pain, we have added psychiatric disorders, substance use disorders, complex medical conditions, and treatment-related issues like medication dependence and failed pain treatments to the biological risk category (Box 1). We also classified these etiological groups as potentially nociplastic or non-nociplastic to align the model with current mechanistic concepts of pain, which are popular in clinical practice and research. Given the long trajectory of chronic pain syndromes like PCT, we also incorporated the life course theory of chronic diseases, which states that chronic diseases like CPS develop from the cumulative impact of biological, psychological, and social stressors throughout the lifespan (77–79), as shown in Box 1.

#### 3.3.6 Pain relief and reward

Most people with pain will naturally seek relief with some pain management intervention (80, 81). However, the experience with the opioid epidemic has shown that repetitive use of pain management for relief can have adverse consequences, including worsening pain and other symptoms (53). So, a comprehensive explanatory model of chronic pain should also account for the adverse impact of repetitively seeking relief.

People often confuse the separate experience of pain relief (removal of distress) with analgesia (the absence or reduction of pain sensation in response to stimulation that would normally be painful). Relief from pain or other distressing symptoms is a non-specific experience mediated by the reward system (53, 82, 83) and is thus mechanistically separate from analgesia, which is mediated by nociceptive systems (53, 82, 83).

Analgesic interventions cannot deliver pain relief without the activation of the reward system (53, 82, 83). In addition, the activation of the reward system can also promote functional improvement and healing behaviors through motivational enhancement (53, 82, 83). Thus, analgesia is neither necessary nor sufficient for pain relief and increased function, and they can be achieved by direct stimulation of the reward system without analgesia (e.g., as with opioids, alcohol, cocaine, etc.) (53). Pain relief by any means is also accompanied by relief from other distressing states like anxiety, anger, depression, constipation, dyspnea, etc. (the whole pain megillah), and vice versa because the same shared reward pathways are involved in all types of relief (53).

**Figure 5:**
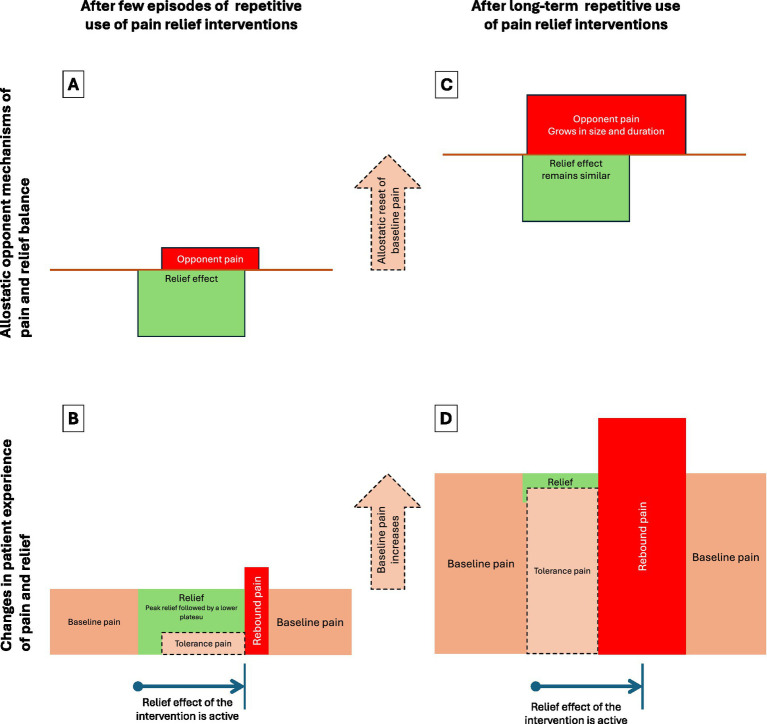
Graphical representation of allostatic opponent effect from the long-term repetitive use of relief interventions and related patient experience of pain and relief. **(A)** Opponent pain effect emerges, beginning and fading shortly after the relief effect starts and ends. **(B)** Due to opponent pain, relief quickly peaks and then plateaus at a lower level. After the relief effect ends, rebound pain briefly exceeds baseline before returning to the prior baseline. **(C)** Opponent effect commences earlier and persists longer at a higher intensity as repetitive use of relief interventions continues long-term. Allostatic mechanisms simultaneously reset the baseline distress to elevated levels. **(D)** Allostatic reset and increased opponent effect raise the patient’s baseline pain and distress and increase the amplitude and duration of rebound/withdrawal pain after each dose, while relief becomes shorter and weaker (tolerance). Relief, though diminished, is still highly salient to the patient.

Pain relief is coded by the body as a reward and is highly valued and evolutionarily preserved because it indicates the removal of a threat to life (53, 82, 83). Pain relief is reinforcing, meaning that patients who are repeatedly exposed to interventions that produce pain relief will develop repetitive seeking of such interventions automatically (53, 84–86). When the body experiences repeated cycles of distress and pain relief, as in long-term pain management, a complex adaptive response called the allostatic opponent effect emerges through adaptations in both reward and anti-reward systems (84–87). The allostatic opponent effect may manifest clinically as an increased requirement for higher doses or more frequent administration of pain management interventions, even as their efficacy and duration diminish (i.e., tolerance). This can create the impression that pain management remains effective, while in fact, chronic pain may paradoxically worsen despite escalation of pain management. The behavioral mechanism of this complex effect is described below and illustrated in Figure 5.

The opponent effect induces pain and distress that commence and subside shortly after relief is initiated and terminated (Figure 5A) (84–87). This leads to reduced efficacy from the same dose and rebound pain once relief concludes. With ongoing administration of relief interventions, the opposing pain and distress intensify, manifest more rapidly after relief initiation, and persist for a longer duration following its cessation. Consequently, the relief achieved with a consistent dose decrease, and a higher dose becomes necessary to attain equivalent relief (tolerance), accompanied by heightened rebound pain post-relief (Figure 5B) (84–87).

With escalating cycles of pain/distress and relief with opponent effect, the neurobehavioral systems that maintain physiological stability also adapt through allostasis (stability through change), whereby the body anticipates repetitive escalation of pain and distress and resets the baseline pain and distress to higher levels to maintain physiological stability and conserve internal resources (Figures 5A,C) (84–87). If left unchecked, the allostatic effect gradually intensifies along with the opponent effect, leading to worsening of baseline pain in addition to decreasing levels of relief with pain management, creating an additional paradoxical pain management-induced chronic pain syndrome (Figures 5B,D). This paradoxical pain is unresponsive to escalation of pain management and often worsens with it (84–87). With a well-established allostatic opponent effect, patients often notice a shift in pain and relief pattern following each dose administration of relief intervention: instead of enjoying lasting relief for the expected duration with each dose, they begin to experience only a brief relief followed by heightened pain during and after the expected period of relief, i.e., an experience dominated by pain instead of relief. This trajectory is consistent with the observation of worsening chronic pain phenotypes among Veterans with a history of mTBI and chronic pain who use excessive amounts of various pain management (2, 3).

The allostatic opponent effect can become harder to reverse with longer duration of repetitive use of pain management, and severe dependence on pain management can set in. This may necessitate the treatment of dependence and tolerance to achieve pain relief in addition to the treatment of the underlying pain condition (84–87). Slow withdrawal or cessation of pain management interventions, a seemingly logical treatment intervention among people with such severe dependence, can often result in protracted withdrawals, beyond the short-lived acute withdrawal, that can cause even worse pain, other symptoms, distress, and disability lasting for months or years (84–87). Thus, both long-term continuation of repetitive pain management and its de-escalation can both cause a paradoxical pain syndrome that is hard to manage and even detect because it mimics the baseline pain syndrome (84–87).

The relief mediated by the reward system and the allostatic opponent effect related paradoxical pain syndrome generated by the repeated pursuit of relief is generally agnostic of the type of distress from which the relief is obtained and the intervention that provides the relief. However, certain types of interventions can be expected to have a lower or higher predilection to generate such paradoxical pain syndromes. For example, addictive substances like opioids and cannabis may be associated with a higher risk of a paradoxical pain syndrome, while non-pharmacological interventions like acupuncture or chiropractic care may be associated with a lower risk. On the other hand, non-pharmacological interventions may be associated with a higher risk of paradoxical pain syndromes among certain people with psychological vulnerabilities for exaggerated addictive responses, and opioids may be used with lower risk among people without such vulnerabilities. More clinical experience and research is required to glean the appropriate duration of each intervention among different groups of individuals that may accrue the benefits of such interventions without significant paradoxical pain syndromes. While waiting for such knowledge to emerge, the management plans for complex chronic pain syndromes like PCT should include clear education and reinforcement of the core concepts of pain relief and its long-term limitations. The choice, number, combination, and duration of interventions should be individualized, and their effects monitored closely for the development of paradoxical pain syndromes. Patients and providers who are not familiar or educated about these concepts may misinterpret the complex paradoxical pain syndrome as a clinical worsening of other painful physical diseases and pursue inappropriate treatments. Another concern is that the allostatic opponent effect creates a desirable illusion that the treatment (e.g., opioid pharmacotherapy) is still “working” even though it is causing more harm and extending barriers to lasting pain relief unknowingly. PCT patients should be advised that these concepts are applicable to repetitive use of any intervention that provides pain relief, such as non-opioid medications, and even benign appearing lifestyle modifications, application of heat and cold and passive interventions like acupuncture and physical manipulations like massage, or physical therapy/chiropractic manipulations (84–86). Taken cumulatively, there is no evidence that long-term repetitive use of analgesic interventions that interrupt or modify nociceptive signals are helpful for chronic pain as a long-term treatment strategy. In fact, long-term pain management of any type is unlikely to achieve *lasting* pain reduction unless patients engage in consistent efforts at functional improvement in the presence of prevailing pain (i.e., self-recovery). Pain management typically provides diminishing benefits beyond about 2–3 months in most situations. Still, pain treatments and symptom management can be provided as a short-term supplement to a self-recovery plan. Understanding chronic pain syndromes like PCT through this recovery lens helps shift the clinical focus from symptom elimination to supporting healing through long-term functional recovery.

#### 3.3.7 Synthesis of an integrated conceptual model- FREIDEM model

The FREIDEM model synthesizes the above six conceptual components and proposes the following:

1. People with chronic pain symptoms mostly have chronic pain syndromes (CPSs) as the diagnosis, where the pain is a separate problem on its own and is not a symptom of a physical disease, even if it started as such a symptom. So, CPSs require evaluation and treatment approaches that are different from those of painful physical disease. Evaluation and treatment of CPS as a physical disease is likely to be ineffective and may perhaps worsen CPS.
2. The pain experience of people with CPS is dominated by nociplastic pain driven by nociplastic biopsychosocial risk factors. Nociceptive and neuropathic pain and triggers are unlikely to contribute much to pain experience among most with CPS.
3. The pain reported by people seeking care for persistent painful conditions is a much broader experience of recuperative debility and distress associated with a complex painful polysymptomatic experience (pain megillah) and not just a simple sensation of pain. This whole pain megillah signals the persistence of recovery response rather than an injury or threat, and is the primary instrument the body uses to initiate, calibrate, and de-escalate the recovery response that enables healing, recovery, and survival from the impact of non-lethal physical and non-physical threats perceived by the body.
4. Thus, a chronic pain condition can be conceptualized as a state of ongoing, interrupted, or stalled recovery from the cumulative impact of exposure to several biopsychosocial threats, mostly nociplastic, over a lifetime. In such stalled recovery states in chronic pain conditions, advancing and accelerating innate self-recovery appears to be the only obvious path to achieve lasting pain relief and improve the quality of life, the primary goals of the patients. Such advancement of self-recovery may require the mitigation of the biopsychosocial impediments to recovery.
5. While short-term symptomatic management of pain and other symptoms that provides brief relief can help with the initiation of the advancement of self-recovery, long-term repetitive pursuit of relief with symptom management often impedes the recovery, due to the automatic development of a paradoxical persistence and worsening of the pain megillah. Situations with such dependence on pain and symptom management with adverse impacts may require the treatment of dependence, including slow deprescribing of it.

**Figure 6:**
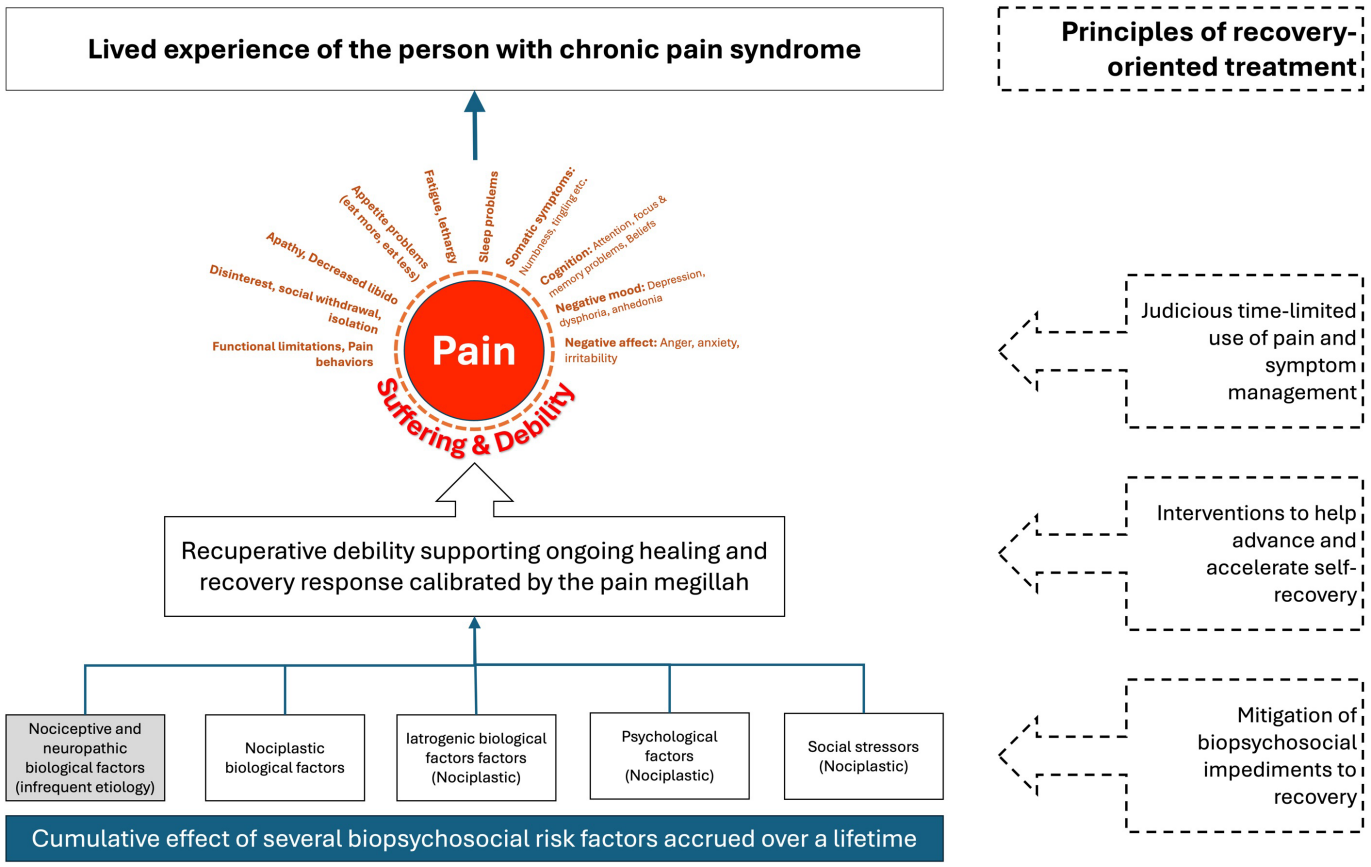
A graphical representation of the conceptual framework of development and persistence of chronic pain syndromes in the FREIDEM model. The persistent, debilitating, and painful polysymptomatic experience (pain megillah) among individuals with chronic pain syndromes represents an ongoing healing and recovery response due to the cumulative impact of several biopsychosocial factors accrued over a lifetime. The pain megillah calibrates the healing and recovery response and abates when sufficient healing is achieved. Recovery-oriented treatment involves interventions that augment and accelerate self-recovery, mitigation of the biopsychosocial impediments to recovery, and judicious use of pain and symptom management in a time-limited fashion.

A graphical representation of FREIDEM model conceptualization is provided in Figure 6.

## 4 From theory to clinical practice with the FREIDEM model

### 4.1 Summary

A summary of the elements of clinical practice based on the FREIDEM model is provided in Box 2 and Figure 6. The goal of treatment is lasting reduction of the distressing pain megillah symptoms and experiences, by therapeutically augmenting stagnant self-recovery with the prevailing pain and distress.

**Box 2 Functional recovery enabling inter-disciplinary evaluation and management (FREIDEM) model for recovering people with PCT.**

***Conceptualization***: Complex chronic pain syndromes indicates a stunted/interrupted recovery state with innate painful recuperative debility in response to the cumulative impact of various biopsychosocial risk factors over a lifetime, not just the initial physical injury.
***Goal of treatment***: Lasting reduction in pain, other symptoms and suffering and improvement of quality of life.
***Overall treatment strategy***: Augmentation and acceleration of innate self-recovery with prevailing pain, mitigation of biopsychosocial impediments to recovery, and limited symptom management of pain.
***Evaluation***: Developing biographical biopsychosocial explanation.

Who is this person? What is the person’s illness problem? How did this person get to this illness state?

- Define the individual experiencing the chronic pain syndrome.
- Define the current illness state beyond pain and debility level.
- Define the trajectory and chronology of the evolution of illness over a lifetime and identify the biopsychosocial factors that contributed at various times.
- Establish the chronic pain diagnosis with chronic primary and/or chronic secondary pain.
- Identify modifiable biopsychosocial impediments to recovery.

***Essential treatment components***

1. A goal-directed plan for self-recovery with prevailing pain and suffering.

- *Options*: Graded exposure therapy, cognitive behavioral therapy, acceptance and commitment therapy, pain reprocessing therapy, cognitive functional therapy, physical therapy, chiropractic care, complimentary integrative care with movement therapy.

2. Mitigation of biopsychosocial impediments to recovery.

- Pain recovery education and coaching to help patients engage better.
- A plan to manage flare ups and psychosocial stressors.
- Lifestyle changes to support recovery.
- Enhance social support for recovery: family, peer, community etc.
- Integrative management of all PCT related components and comorbidities.

- Nociplastic comorbidities like psychiatric diseases, substance use and use disorders, distressing medical disorders, iatrogenic augmenters like medication dependence, polypharmacy, and overuse of pain management, procedures and surgeries for pain.
- Non-nociplastic comorbidities: Musculoskeletal disorders, systemic inflammatory diseases, cancer, post-surgical states, visceral conditions, neuropathic conditions, etc. if indicated

3. Judicious use of pain management and other symptom management.

- Limit symptom management of pain and other symptoms for a short time (8–12 weeks) in select cases to initiate self-recovery.
- Avoid and/or wean off long-term symptom management of pain and non-pain symptoms.

**Figure 7:**
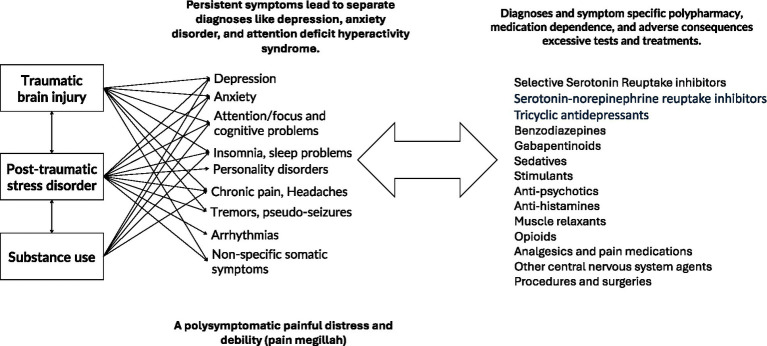
Shared symptoms of traumatic brain injury, post-traumatic stress disorder, substance use for self-management, and the consequences of symptom management can create a complex polysymptomatic chronic pain syndrome.

The FREIDEM model evaluation aims to understand the details of functional limitations, assess patient symptom load, ascertain an IASP classification-based diagnosis, and identify biopsychosocial impediments to recovery from a narrative summarization of the patient’s life with pain and other symptoms. Treatment is formulated based on the level and nature of the debility and impediments identified during evaluation. Therapeutic mitigation should address functional recovery and biopsychosocial impediments to self-recovery, with supportive management of pain and other symptoms. The shared symptoms of mTBI, PTSD, and chronic pain, and the consequences of symptom management, can create a complex clinical scenario that can overwhelm both providers and patients (Figure 7).

The FREIDEM model proposes that treatment for complex chronic pain syndromes like PCT involves escalating functional activity with the prevailing symptom load to achieve healing and recovery, i.e., improving psychosocial life with the symptoms of mTBI, psychological trauma, and comorbidities. Recovery in PCT may correlate with improvement of symptoms, but recovery in PCT is primarily concerned with living well through healthy adaptations to the experience following polytrauma events.

### 4.2 Clinical practice

#### 4.2.1 Case example

A detailed evaluation of an archetypal patient is provided in Appendix 1, and a concise summary is provided below.

A 50-year-old US military Veteran suffered combat trauma without major injuries and subsequently developed low back pain, PTSD, and several symptoms of mTBI. He used alcohol and cannabis heavily to cope with both pain and PTSD. However, his conditions worsened and led to military discharge, followed by lifelong struggles with loss of military identity and employability. Later, he developed progressively worsening pain at multiple sites with profound suffering and disability that worsened even further after he quit alcohol and cannabis in his early 40s, after he went through a mental health crisis that required hospitalization. Then came knee buckling and fear of movement, with dependence on assistive devices. He also underwent several pain management courses and surgeries; however, pain seems to have worsened after each surgery and unsuccessful pain management course. He was also on opioids for a decade but was tapered off a few years back with worsening pain and debility. His PTSD, depression, and anxiety remained uncontrolled, along with pain and debility, despite mental health treatments. By his early 50s, he was unemployed, on disability benefit, divorced, and having family, financial, and social difficulties. His pain seems to have worsened following similar major life stressors throughout his life. Based on the information he received from his prior healthcare providers, he believes that he has an incurable disease that will worsen progressively. He had chronic post-mTBI symptoms like headaches and memory/focus problems from the original combat trauma that also worsened along with the other pains despite a series of specialist treatments and compromised his function significantly.

#### 4.2.2 Evaluation

The patient evaluation in the FREIDEM model involves systematically asking the following questions: Who is this person with PCT? How is this person’s life affected by the pain megillah and PCT components, such as PTSD? How did this person get to this state of complex debility with pain? What factors contributed to the progression to this illness state? This approach is consistent with Narrative-Based Medicine (NBM) practice (88–92). The NBM practice is based on the realization that narratives are the essential tool by which we make sense of the world and communicate with each other about such problems (88–92). A detailed discussion regarding NBM practice can be obtained elsewhere (88–94).

In the FREIDEM model, the provider collects patient perspectives and organizes them into a chronological narrative that describes the roles of different biopsychosocial factors in the progression of the chronic pain condition, using the scientific framework of the FREIDEM model. A systematic approach is undertaken, first defining the impact of chronic pain, followed by creating the individual biographical narrative of the evolution of the pain syndrome, treatments, social history, psychiatric history, substance use history, and medical history. The progression of the pain syndrome is then linked to the chronological history to create a comprehensive biopsychosocial narrative like the one provided above. A biopsychosocial explanation is then created from this narrative using the etiological framework of the FREIDEM model. In this process, the provider employs the simple logic of sequencing events in any medical history creation, parsing what happened in the biopsychosocial realms in relation to the progression of the pain syndrome to assign causal connections (i.e., “this” happened in the biopsychosocial realms and then, “that” happened in pain and function realm, so, what happened in biopsychosocial realms explains what happened in the pain and function realm based on the BPS model)

#### 4.2.3 Diagnosis of chronic primary and secondary pain

The same patient with a complex chronic pain syndrome like PCT can have several component chronic pain diagnoses, including both chronic primary pain and chronic secondary pain syndromes. A detailed discussion of this diagnostic process is beyond the scope of this manuscript, and details can be obtained elsewhere (17). We provide a simplified flow chart for diagnosis in Appendix 2.

In practice, people with complex CPS like PCT can have several chronic pain diagnoses, including chronic secondary musculoskeletal pain, chronic primary widespread pain syndrome, and chronic secondary (post-traumatic) headache syndrome. Although it is important to classify and characterize the component chronic pain syndromes, treatment prioritizing functional recovery is required for resolving both chronic primary and secondary pain impacts (17). So, irrespective of the CPS diagnostic categories involved, recovery among people with complex CPS, like PCT, must be supported with appropriate integrative management of post-TBI sequelae, PTSD, comorbidities, and other biopsychosocial impediments. Management of life and recovery with chronic headaches and attention/cognitive limitations may be particularly challenging issues.

### 4.3 Comprehensive treatment of chronic pain

The three components of comprehensive chronic pain treatment are discussed in detail below.

#### 4.3.1 Goal-directed plan for self-recovery

Relief from pain and other distressing symptoms (pain megillah) is unlikely to be achieved without escalation of functional activities with the prevailing pain and other distressing symptoms, including “TBI symptoms.” Providers and others can only play a supportive role in this intervention. Most people presenting with chronic pain will have attempted to pursue this strategy aggressively (“fighting through pain”) without success before they seek treatment. In such people, recovery stalls not because of a lack of desire or effort to get better, but despite their intense desire and extensive prior efforts. It is incredibly challenging for patients to consciously engage in potentially painful or distressing therapeutic activities when the subconscious innate healing and survival motivations remain in opposition. Therefore, patients need a formal program with specific time-bound goals (e.g., “I will be able to walk a mile in 3 months without much difficulty”). Goals need to be implementable in daily life (not just with clinician support inside the clinic). Evidence-based treatment strategies pertinent to biopsychosocial activation with prevailing pain include cognitive behavioral therapy-pain, cognitive functional therapy, pain reprocessing therapy, and graded exposure therapy in conjunction with physical therapy. These therapies provide directed education and functional training for 8–12 weeks, after which patients can typically continue their recovery independently. Less evidence-based treatments like chiropractic care, massage therapy, and acupuncture can be refashioned into functional recovery programs so long as they help the patient prioritize activity escalation with pain. However, profoundly debilitated patients may benefit from inpatient rehabilitation care to start their recovery.

#### 4.3.2 Mitigation of biopsychosocial impediments to recovery

There are several biopsychosocial impediments to recovery, and the most important ones are addressed below.

*Education to remedy inadequate actionable knowledge:* Self-recovery is possible only if the person with pain is ready and willing to engage in painful recovery activities. Patients often must unlearn their existing beliefs and treatment approaches and learn new information to engage in recovery treatment effectively. The concepts of chronic pain, biopsychosocial etiological model, recovery-first treatment approach, and deemphasizing the traditional pain management strategy may appear complex and confusing to many patients. Therefore, they will require ongoing education and coaching to maintain their engagement in self-recovery. Providers should be tolerant of fluctuations in patients’ beliefs. The educational content should be accessible to laypeople without complex neurobiological jargon, with a focus on action-based learning.

*Managing chronic pain flare-ups:* A chronic pain flare is defined as a worsening of the pain experience that lasts from hours to weeks, which is difficult to tolerate and impacts activities and/or emotions (95). A chronic pain flare is distinct from normal daily fluctuations in pain intensity and new acute pain from unrelated injuries or diseases, and is dominated by increasing non-pain non-physical symptoms like depression, anxiety, and fatigue rather than increased pain intensity (96–98). However, most patients attribute flare-ups to physical reasons like overactivity, static posture, biomechanical dysfunction (such as damage to the spine), or certain medications (99). On detailed inquiry, most patients report that flares that are dominated by non-pain symptoms were indeed triggered by unpleasant non-physical experiences, such as stressful situations (family, marital, employment, financial, trauma reexperiencing, PTSD worsening, etc.), and doing ordinary activities in life, like mowing the lawn or cleaning the kitchen, which is characterized as overactivity. Higher frequency and severity of pain flares significantly restrict function (100, 101) and are often the most common reason for lack of progress. Pain flares typically resolve on their own, although the resolution can sometimes be prolonged and difficult, and may involve increasing non-pain experiences (95). Such experiences should be normalized as part of the natural cycles of chronic pain, removing the fear of them. It is important to highlight the relationship between pain flares and stressful social events, which are unavoidable in routine life. Patients should be coached to look for non-physical explanations and not to seek escalation of provider-based care, which tends to make the flares more severe and longer-lasting. Patients should be coached not to be fearful of overexertion-induced flares, as they are not indicative of injuries and usually self-terminate in about 1 day. Still, patients should avoid overexertion in the therapeutic self-recovery activities. Flare-ups tend to decrease in frequency and severity as function increases.

*Lifestyle changes to support recovery:* Many of the patients have lifestyles dictated by pain-related avoidance behaviors and pain management needs. They will need coaching on constructing daily life routines consistent with recovery. This includes changes in the schedules of daily mundane activities, such as getting up in the morning, going to sleep, and eating three meals at specified times, as well as changes in the content of their food and in their physical activity.

*Enhance social support for recovery:* People engaged in chronic pain recovery may need a lot of support but often lack such support systems. Therefore, it is essential to coach them on rebuilding these social support systems that foster recovery. Family members, friends, peer support, and community resources can all be leveraged to expand the patient’s social support system.

*Integrative management of comorbidities*: Multimorbidity is common in patients with complex CPS like PCT patients, and it is essential to manage multimorbidity in an integrative, comprehensive manner and to avoid care from separate, siloed providers. Special attention should be paid to the management and prevention of iatrogenic augmenters like medication dependence, polypharmacy, and inappropriate use and failure of pain management and surgeries. Given the multimorbid nature of complex CPS such as PCT, providers should become proficient in the careful, parsimonious use of interventions to avoid iatrogenic worsening of CPS. Non-nociplastic risk factors can occasionally drive nociplastic pain through bottom-up mechanisms in addition to top-down central nociplastic mechanisms (102, 103). For example, the chronic knee pain with nociplastic features in a patient with a chronic widespread primary pain diagnosis may resolve with a knee replacement. In chronic secondary pain syndromes, the clinician and patient must also assess whether non-nociplastic risk factors are the key drivers and, if so, aggressively manage them. Care should be de-escalated if the patient is unresponsive or if non-nociceptive factors are deemed not significant contributors at that time.

#### 4.3.3 Judicious use of pain and symptom management

In most instances, symptom management of pain and other symptoms should ideally be used for only 8–12 weeks to help initiate or accelerate recovery. Patients should be coached to begin their recovery while their distress is under control. Patients should be enabled to continue self-recovery regardless of whether intervention continues. However, antidepressants, gabapentinoids, and opioids used for refractory chronic pain may require a longer duration of use. They should be discontinued when the patient achieves reasonable functional improvement or when treatment effectiveness is lost. While some people can engage in distressing and painful recovery activities without any pain or symptom management interventions, others may require a longer duration or several types of pain and symptom management. The care should be individualized based on the patient’s need for support of recovery, balancing the benefits with the harms, including the potential for paradoxical pain syndrome and unhelpful dependence.

#### 4.3.4 Monitoring the progress

CPSs like PCT are complex illnesses. So, comprehensive monitoring of the various aspects of the individual’s function is essential to guide treatment. We recommend a comprehensive measure like Pain Outcome Questionnaire (POQ) or Pain, Enjoyment of life and General activity (PEG) questionnaire along with Short Form-12 (SF-12) to monitor the progress of the overall functional experience. Pain intensity measures should not be the primary tools for measuring progress, as pain subsides only after stable functional recovery. It is often helpful to set individualized goals for the patient to achieve- for example, we would like the POQ to decline from 110 to 80 or the PEG from 10 to 8. Many patients with complex CPSs present with disability for several years or even decades. The objective measures might not show much change even when they report they are doing better in their life. Measurements of pain-specific psychological factors, such as the Pain Self-Efficacy Questionnaire and the Pain Catastrophizing Index, are also useful. Comorbidities like depression, anxiety, and PTSD may also be monitored using objective measures if they are contributing to the CPS of the individual. While objective measurements are important, narrative descriptions of the functional state of the individual is critical for both providers and the patients to make sense of the progress or lack of it. This is consistent with the NBM methodology of patient evaluation detailed in Section 4.2.2.

#### 4.3.5 Distinction of the FREIDEM model from conventional approaches

The FREIDEM model’s simple design and articulation of complex care plan often create a misconception that most of its elements are already part of standard clinical practice, requiring only minor adjustments from healthcare providers. However, there are several stark differences between clinical practice based on traditional models and the FREIDEM model, some of which are highlighted below. Conventional approaches, whether applied in isolated or multidisciplinary practice, are typically grounded in the assumption that chronic pain signals the persistence of a physical threat like injury or disease (imagined or real) affecting the pain site and/or a protective response against such threats that may be enhanced by other BPS factors. In contrast, in the FREIDEM model, persistent pain (pain megillah) is conceptualized as a tool the body uses to foster recuperative debility that advances healing and recovery, albeit unsuccessfully, among people with CPS due to the cumulative impact of several BPS factors. Consequently, these represent fundamentally different paradigms, necessitating substantial changes in clinical practice to use the FREIDEM model. These differences in evaluation and treatment become clear when the previous treatment of the person in the earlier case example is compared to the plan generated based on the FREIDEM model. For instance, the individual received multiple diagnoses of physical illnesses, many of them incurable, as explanations for chronic pain and other symptoms. This led to several courses of unsuccessful pain and symptom management as well as surgical interventions intended to be curative. The current ICD-11 diagnostic criteria and conceptualization of CPS (pain is a problem on its own and not a symptom of a physical disease at the pain site) were not used as an explanation for his chronic pain and other symptoms, and to guide treatment. In addition, there was no significant attention paid to the nociplastic BPS factors with regards to etiological explanation or treatment. In contrast, the FREIDEM model-based evaluation ascertained ICD-11 CPS diagnoses and provides a detailed BPS explanation that anchors further treatment (see Appendix 2 for details). While most, but not all, of the traditional models focus on long-term pain and symptom management as the primary focus of treatment, FREIDEM model-based care advises against such an approach, and deprescribing of such care is an important therapeutic intervention because of pain management-induced pain syndromes. In contrast to traditional models, the FREIDEM model encourages the achievement of functional improvement with the prevailing load of pain and distressing symptoms instead of insisting on control of pain and distress as a prerequisite for recovery. The FREIDEM model’s principle that lasting pain relief follows functional recovery might seem like a minor difference from traditional models, but it represents a 180-degree shift in actual clinical practice. In addition, the limitation in physical function is seen as an (mal) adaptive motivational state that is treated with cognitive behavioral interventions (graded exposures with relaxation) and not as a structural physical deficit to be treated with traditional interventions targeting muscle strengthening and joint alignment, along with safety-assuring interventions like assistive devices. In traditional models, nociplastic biological factors like psychiatric and substance use disorders and psychosocial distress are usually considered as separate clinical issues to be managed separately, whereas in the FREIDEM model, they are considered as key etiological drivers and hence primary targets for the remedial treatment for CPS. In summary, FREIDEM model-based practice is a significant paradigm shift in clinical practice related to complex CPS like PCT.

## 5 Limitations and future directions

The close parallels between chronic pain syndromes and PCT suggest that the FREIDEM model approach may yield benefits for PCT patients, particularly patients with treatment-refractory PCT or those presenting with pain as a primary symptom. However, several issues need to be addressed before validating a FREIDEM model approach to PCT as the standard of care. Further research efforts are needed to assess whether a practice based on the FREIDEM model results in lasting positive outcomes for patients with PCT. Cognitive impairment following TBI may create challenges for patient engagement with complex disease and treatment. In response, model modification may be required to address PCT-specific challenges, such as recommendations for patients who experience significant affective or cognitive changes following mTBI.

### 5.1 Implementation challenges to be overcome

Implementing the FREIDEM model marks a major shift from current clinical practice models used in CPS, This transition presents numerous challenges, several of which are discussed below. Adopting the FREIDEM model requires providers, clinics, and health systems to embrace a significant cultural change in pain management - moving away from the traditional but scientifically flawed practice of treating CPS as symptoms of other physical illnesses and instead approaching CPS as distinct complex biopsychosocial entities. Achieving this shift demands considerable effort from institutional leaders, healthcare providers, patients, and other stakeholders.

Currently, there is likely an insufficient workforce trained in the interdisciplinary concepts and management of CPS, making such changes in established care cultures particularly challenging. Therefore, it is essential to establish clear pathways for both initial and ongoing training and mentorship. The FREIDEM model can be implemented either with a single trained prescribing provider (such as a physician or midlevel provider) paired with a nurse, or through an interdisciplinary team. Regardless of the structure, care addressing primary drivers of CPS - like psychiatric disorders, substance use, complex medical conditions, iatrogenic issues, and psychosocial distress - should predominantly occur within the clinic or remain under close supervision if managed collaboratively with other clinics. If clinics maintain siloed, traditional multidisciplinary care models where providers follow differing approaches, confusion and suboptimal care are likely outcomes. Additionally, payment structures for the time-intensive care required by the FREIDEM model pose challenges for privately insured populations, and allocating staff for intensive yet lower-volume outpatient care can be difficult even for integrated systems like Veteran Health Administration. Thus, there is also a need to develop sustainable business models to support this type of care. These are not insurmountable barriers but merely challenges to be overcome to develop comprehensive care programs for extremely vulnerable patients with complex CPS like PCT.

## 6 Conclusion

Recent clinical advances in conceptualizing and treating chronic pain syndromes have yielded benefits but have not been applied to PCT care. By conceptualizing PCT as a complex chronic pain syndrome, this paper evaluated the potential merits of a FREIDEM model approach to PCT care. The FREIDEM model offers an integrative biopsychosocial approach to personalized illness conceptualization, clinical diagnostic evaluation, and multimodal treatment. Further efforts are needed to assess whether a practice based on FREIDEM model results in positive outcomes for patients with PCT. The FREIDEM model offers an opportunity for long-overdue conversations around the benefits and limitations of person-centered transdisciplinary care for people with PCT and other complex chronic pain syndromes.

## Data Availability

Raw data supporting this article are unavailable because they contain sensitive, identifying patient information. Further inquiries can be directed to the corresponding author.
