# Supplementary material for: Conceptualizing and treating the polytrauma clinical triad as a complex chronic pain syndrome
Source: Front Neurol. 2026 Jul 17;17:1752206. doi: 10.3389/fneur.2026.1752206 (PMC13423984; doi:10.3389/fneur.2026.1752206)
Supplement: Supplementary file 1 [file Supplementary_file_1.docx]

**Appendix 1: Detailed history of a Veteran with a complex chronic pain syndrome, evaluation and development of comprehensive treatment plan based a biopsychosocial explanation as per the Functional Recovery Enabling Inter-Disciplinary Evaluation and Management (FREIDEM) model.**

**Reason for Consultation:** Chronic back pain and bilateral knee pain uncontrolled.

**Description current state:**

**Pain sites:** Low back, mid-back, knee bilateral, ankles bilateral, feet bilateral, legs bilateral, hips bilateral, shoulders bilateral, neck, and headache.

**Other symptoms:** Anger, irritability, anxiety, depressed mood, difficulty concentrating and attention/focus problems, brain fog, poor sleep, nightmares, tinnitus, fatigue, and lethargy. All these symptoms have been present for as long as he can remember, along with pain.

**Pain scores:**

Pain intensity: 8/10, Pain related distress- 10/10, Pain-related interference in daily activities- 10/10.

Number of sites of pain= 16; Fatigue (0-3)= 3; Trouble thinking or remembering (0-3) = 3; Waking up tired or unrefreshed= 3; Headaches = Yes; Depression= Yes; Pain in lower abdomen= No

**Description of functional limitations:** He is largely confined to home except for doctor visits and uses walker or wheelchair for mobility. He goes shopping once a week or every two weeks. Rarely visits family or friends. He can get out of the house and do shopping and essential things on a good day and in bed all-day except for toileting and eating on a bad day. He has about 20 bad days a month. Mornings are the hardest and he can move better as the day progresses. He reports that he can push through pain and still do things that he cannot avoid. The other day, he went to see his grandson play football. It was hard and he was in bed for the next week due to a pain flare. He generally avoids people and situations where he must interact with a lot of people. He feels safe at home.

**Pain flares:** He gets at least 2 flares a month which confines him to the bed. Pain is nearly the same, but he cannot move due to stiffness, and it gets really depressing. He rests and it resolves in 1-2 weeks. He used to go to emergency rooms and doctors before but do not anymore as nothing seems to help. He reports that change in weather, stress and overactivity triggers the flares frequently. Sometimes, it happens for no reason.

**Leg buckling and falls:** He used to have a leg buckling and fall few times a week). It is less now since he started using walker and wheel-chair. It still buckles but he can hold on without falling. He reports that his leg buckling is preceded by a sharp shooting pain in the back or down the legs, and his legs just gives out. He can get up and walk in a few minutes. This happens when he is tired after walking for a while. He avoids steps and walking longer distance because of fear of falling.

**Summary of the problems:** Severe debility and emotional distress with chronic multi-site pain, several other CNS symptoms, leg buckling, fear of falls and fear of activities that fluctuates with activity, emotional stress and weather change.

**Detailed pain history:**

His low back pain started at 22 years after a motor vehicle accident while serving in in the military. His military vehicle rolled over in an IED explosion while he was deployed in Iraq. He report being passed out for about a minute after hitting his head. He did not suffer major injuries and was back to work next day. Slowly, this back pain got worse, and he started having leg pains. An MRI was done and he was told that he had disc prolapses. He was soon unable to perform his military duties. He tried several pain management options in the military, but none of them helped. So, he underwent a back surgery when he was 25 years. Things improved for about 6 months, but pain became worse, and he was again restricted from his duties mainly due to back pain. He also started developing migraine headaches following the accident which slowly got worse. Towards the end of his military career, he was also struggling with memory and focus issues that impeded his work at times. He was medically discharged from the military when he was 26 years old.

He started working in different jobs during his civilian life with the help of pain management. However, he still had to take a lot of sick days. He slowly developed pain in knees, ankles, neck, feet, and hips in addition to his back pain and headaches. He was told that he had progressive arthritis. He was eventually started on opioid pain medications when he 32 years after all other pain medications, PT and other pain management were unsuccessful. He lost his job around this time because of his absence due to pain but soon found another one with less physical work. Opioids were helpful for a while, but all these pains worsened, and his physical function deteriorated along even with increase in opioid dose and additional pain medications.

He lost his second job around 40 years of age with extensive sick leave use due to pain. He underwent a second back surgery around this time. This helped for a few months, but his pain worsened even further after this. He was also going through a lot of stress from financial problems, family problems and mental health issues during this time. It was a difficult time. He soon found another desk job and was able to maintain work with the help of increased opioid dose and other pain management. By this time, his headaches had worsened and his struggles with memory and focus worsened.

Soon, he started developing knee buckling and falls. He started using cane because he was afraid of falling. He started avoiding activities that precipitated knee buckling like using stairs and walking more than a block or two. He had a left knee surgery when he was 44 years, but this did not help with pain or knee buckling. By 46 years, he was severely disabled from pain and deterioration of his overall health including mental health despite being on several strong medications that sedated him. He stopped working and was on disability when he was 48 years. His leg buckling progressed despite another right knee surgery and he started using walker instead of cane, and wheelchair for longer distances. He was spending most of his time in recliner and bed by this time. Cooking and self-care became challenging. His shoulder pain worsened, and he underwent a left shoulder surgery around this time without much benefit.

His pain and debility progressed despite all these treatments. In his late forties, he had another left knee surgery and right shoulder surgery without any benefits. Around this time, his doctor started tapering his opioids and this was stopped about a year back. His pain, debility and mental health deteriorated considerably, and he underwent another back surgery about 6 months back with no benefits. His pain and debility instead worsened after this, and he is now practically confined to his home. Over the years, several doctors told him that he has incurable and progressive arthritis and disc disease that is causing this level of pain. He has numerous X-rays and MRIs over the several decades.

**Opioids history:** Opioids were started at 32 years as oxycodone 5 MG TID and slowly increased to oxycontin 60 MG BID and oxycodone 10 MG QID by 40 years of age. Doing poorly on it. The PCP tapered it off slowly a year back. His pain, other symptoms and debility worsened. On opioids, he was at least able to do a few chores at home and take care of himself. Now, he is confined to his recliner and bed most ties. He is not going out at all. He needs help but pushes on without it.

**Current medications:** Gabapentin, duloxetine, amitriptyline and trazadone. Not working.

**Other Medications tried in the past-** Several types of non-steroidal anti-inflammatory agents, several topical agents, and over the counter medications and supplements.

**Non-pharmacological treatments-** Chiropractic care, PT and acupuncture several times. Psychological treatment once.

**Procedures:** Several courses of spinal and knee injections- only brief relief. RF ablation in the back.

**Surgeries for pain-** Failed back (x3), shoulder (x2) and knee (x3) surgeries. He was evaluated by a neurosurgeon and orthopedic surgeon recently. No surgical options.

**X-rays and MRIs:** MRIs of neck, lumbar spine, shoulders and knees available. These show age related degenerative changes and post-surgical changes. No pathologies that require immediate surgical attention reported.

**Social history:**

He had a happy childhood. He grew up with 2 siblings in a happy home with both his parents. They were not rich but did not lack anything in their lives. He joined military at 19 years and loved it. He was planning to retire after 20 years of service. He was medically discharged at 26 years. He struggled with his civilian life and felt cheated out of his life. He tried several jobs and had significant challenges staying employed due to his chronic pain. He lost his first job in mid 30s but got another one quickly. He got married in his early 30s. His wife was initially understanding regarding his struggles with chronic pain and mental health. He had two children. They are grown up and doing well with grandchildren.

His life started falling apart with mental illness in his 40s. He stopped drinking and cannabis use in his early 40s. Things improved for a while, but mental illness and pain became too difficult to handle as he got more disabled, and his marital relationship suffered greatly. He lost his second job in early 40s and it took a while to find a new one. His wife left him in his late 40s. Around this time, he lost his third job due to extended absences and he has been unemployed since then. By 48 years, he was on social security disability benefits, and he could barely survive with added VA benefits. He was always under severe financial stress and this escalated.

He has been living alone and isolated since his wife left him. He is not keeping up with his friends or family- “nobody wants to hear about my troubles.” He is also dependent on walker and wheelchair for mobility. He is depressed about it and wondering how a very active man ended up in this hopeless situation.

**Psychiatric history:**

He was in good health till he suffered the IED explosion in military while posted abroad. He was also exposed to several violent combat incidents that distressed him greatly. He soon started experiencing nightmares, flash backs, sleep problems, jitteriness, anxiety, depression and panic attacks. But he fought through it because he wanted to continue his military career. These symptoms worsened after his military discharge and continued to worsen in his 30s. He stared drinking heavily while in military to cope and he added significant use of cannabis soon. He was treated for depression and anxiety with several medications. He was finally diagnosed with PTSD after a hospitalization for a suicidal attempt when he was 41 years. He received some group therapy and medications. However, PTSD continued to worsen, especially after he stopped alcohol and cannabis with professional help when he was 42 years old. He currently isolates at home because that is the only place he feels safe.

**Substance use history:**

He started weekend binge drinking alcohol in military in his early 20s. This escalated to daily drinking after the traumatic IED with 6-7 drinks at night till he was knocked off to sleep. He regular regular cannabis use around his military discharge time. Cannabis and alcohol use escalated after military discharge. But he was able to maintain a job and his family life with it. However, soon, his mental state got steadily worse that he started missing in his work and life. He went through severe mental health crisis around 40 years that required hospitalization. So, he decided to quit alcohol and cannabis. He did so at 42 years with professional counseling. He thought his mental state and overall health would improve but it has worsened after quitting substance use. However, he has no desire to go back to his drinking and cannabis days. He is glad that he quit it.

**Medical history:**

In his 40s, he was diagnosed with mild traumatic brain injury (mTBI) from the IED explosion in his 20s but was advised that he did not need any treatment for it. The TBI clinic told him that his memory and focus issues were related to his psychiatric condition and he was referred to his psychiatrist. He received extensive treatments for his headaches thought to be a sequalae of mTBI, but his headaches worsened over the years. His chronic headaches were treated with several headache medicines by several headache specialists without any lasting relief. So, he stopped all these medications. He reports that opioids provided him with the best relief and his headaches, focus, and memory problems worsened dramatically after opioids were stopped. Around the age of 50, he was struggling significantly with his memory and focus issues to the extend that he had to maintain a reminder system to do even everyday chores. Neuropsychological evaluations were within normal parameters though.

He has mild diabetes and hypertension that are controlled with medications. He is overweight. No other major medical illnesses are reported.

**Focused physical examination**

Pain behaviors: Lots of sighing and some groaning when shifting in seat and moving.

Gait: Walks leaning on the walker and very guarded, slow and deliberate in movements.

Sitting down and standing up in chair is guarded with breath holding and groaning.

Observed walking short distance without walker. No physical deficits observed.

Overall examination did not reveal any focal deficits in power.

Other examinations were normal.

**Mental status:**

Alert, oriented in time, place and person. Conversation is coherent, goal oriented and future focused. He is able to pay attention to the information presented and process it well.

Mood: somewhat depressed mood and sense of despondence around pain “I don’t know how I could live like this?”. No suicidal or homicidal ideations endorsed.

Affect: Somewhat anxious, angry and irritated disposition. Congruent with mood and cognition around pain.

No symptoms suggestive of mania or psychosis.

**Laboratory:**

Urine Drug screen- negative for any substance

Liver function test: Normal

Other labs: Unremarkable.

**Prescription Drug Monitoring program (PDMP) review:** No aberrancies noted

**Concise chronological summary of events**

In his early 20s, he suffered combat trauma without major injuries and subsequently developed low back pain, PTSD and several symptoms of mTBI. He used alcohol and cannabis heavily to cope with both pain and PTSD. However, his conditions worsened and led to military discharge followed by life-long struggles with loss of military identity and employability. Later, he developed progressively worsening pain at multiple sites with profound suffering and disability that worsened even further after he quit alcohol and cannabis in his early 40s after he went through a mental health crisis that required hospitalization. Then came knee buckling and fear of movement with dependence on assist devices. He also underwent several pain management courses and surgeries; however, pain seems to have worsened after each surgery and unsuccessful pain management course. He was also on opioids for a decade but was tapered off few years back with worsening pain and debility. His PTSD, depression and anxiety remained uncontrolled along with pain and debility despite mental health treatments. By his early 50s, he was unemployed, on disability benefit, divorced and having family, financial and social difficulties. His pain seems to have worsened following similar major life stressors throughout his life. Based on the information he received from his prior healthcare providers, he believes that he has an incurable disease that will worsen progressively. He had chronic post mTBI symptoms like headaches and memory/focus problems from the original combat trauma that also worsened along with the other pains despite a series of specialists treatments and compromised his function significantly.

**Main problem:**

Severe debility and emotional distress with chronic multi-site pain, several other CNS symptoms, leg buckling, fear of falls and fear of activities that fluctuates with activity, emotional stress and weather change. All these problems progressed over decades.

**Chronic pain diagnosis:**

1. Chronic widespread primary pain syndrome
2. Chronic post-surgical pain syndromes
3. Chronic post-traumatic headache

**Chronological biopsychosocial explanation**

This patient has a complex chronic pain syndrome that developed and progressed initially in association with PTSD, mTBI, severe alcohol and cannabis use and distress and loss of identity with military discharge. This worsened with stress associated with employability, financial stability and family life compounded by worsening PTSD and escalating alcohol and cannabis use. This pain syndrome worsened further with protracted withdrawal syndrome from cessation of alcohol and cannabis use and subsequent escalation of PTSD symptoms despite treatment. Failure of several inappropriate treatments based on a diagnosis of chronic secondary pain syndrome (pain management and surgeries) including complex persistent opioid dependence, polypharmacy with dependence, protracted withdrawal syndrome from opioid cessation and a persistent belief that this pain syndrome is driven solely by incurable medical conditions were also significant contributors to the pain syndrome over past 3 decades. Several pain specific psychobehavioral factors like fear avoidance behaviors, pain catastrophization, loss of self-efficacy and fear of falls also seem to have contributed greatly. Several social stressors like loss of military identity, social roles, employment, and financial stability and relationships, lack of support system, and loss of key family relationships are likely critical drivers of this pain syndrome. It is unlikely tat this pain syndrome for this patient is driven by nociceptive or neuropathic etiologies like arthritis, vertebral disc disease or pinched nerves.

**List of major factors contributing to the chronic pain syndromes**

- PTSD
- Alcohol & Cannabis use disorder in remission
- Protracted withdrawals following SUD remission
- Complex persistent opioid dependence with protracted withdrawal syndrome following opioid cessation.
- Polypharmacy
- Overuse of pain management despite multiple treatment failures
- Maladaptive beliefs about cause of pain like incurable musculoskeletal diseases
- Fear avoidance behaviors, pain catastrophization, loss of self-efficacy
- Knee buckling, fear of falls and assist device dependence
- Series of social stressors including loss of employability, self-identity, relationships and support system.

**Comprehensive treatment plan**

**Rehabilitation plan**

1. **Acceptance of biopsychosocial explanation and self-recovery with pain:** The patient was educated that rehabilitating himself with pain, with assistance of providers, is the only path to durable pain reduction. His chances of successful engagement in self-rehabilitation would be low without the acceptance of BPS explanation and the rejection of the notion that he has incurable disease. He has moderate acceptance in these areas but struggles to accept that he has no incurable progressive physical disorders. We will continue the education and conversation. Reassured that he has no incurable diseases and the findings on X-rays and MRIs are real but not contributing to his chronic pain.
2. **Self-rehabilitation plan:** Fear avoidance behavior and graded exposure to painful activities with relaxation was explained. He verbally confirmed understanding. He agreed to start walking 10 feet outside the home without assist devices and stand 5 minutes to kitchen chores twice daily as graded exposure and escalate it every 2 weeks. A 3-month goal of walking 2 blocks without assist device and 15 minutes of continuous standing for kitchen chores was established. He was provided with education regarding stretching the hamstrings and mid-back muscles daily.
3. **Plan to function well with Pain Flares:** He was provided detailed education and advice to manage pain flares. The following were explained:
   - Pain flares are mostly flares of non-pain symptoms & debility and not pain.
   - Flares are ‘normal’ expected events in chronic pain and not new acute injuries. Flares occur mostly due to non-injury factors like weather change, stress, emotions, etc. For an explanation, look at what has changed in life and not what could be found on X-rays or MRIs
   - Flares are self-remitting, and no treatment needed. Physician and ER visits, X-rays and MRIs often worsens pain/disability and delays resolution. Short rest and early mobilization with pain will result in improvement.
   - Overactivity pain is normal (is not due to new injury) and everyone gets it. People with chronic pain have a lower threshold for overactivity pain compared to people without chronic pain. Don’t fear it or panic, expect it to occur if you do a higher level of activities than normal. Like every time before, it will resolve after a brief rest.
   - Specific behavioral interventions for knee buckling to be introduced during the next visit.
4. **Plan to function well with stress**: Explained the role of stress in development of chronic pain and pain flares. Will introduce mindfulness-based interventions to function with stress in the next visits.
5. **Comorbidity management plan**
   - **PTSD-** He has significant fear avoidance behaviors related to PTSD that limits his function and ability to participate in rehabilitation activities. He also has significant nightmares with poor sleep and emotional dysregulation.
     - - Provided basic education about PTSD and its treatment.
       - Referred to Prolonged Exposure Therapy, but patient could not tolerate.
       - Started on home-based non-trauma in-vivo graded exposure with relaxation- For example, expose to a crowded store, experience the anxiety and relax the body through deep breathing and continue to walk around in the store while experiencing anxiety and no trying to control it. Escalate the duration of exposure every 2 weeks.
       - Change from duloxetine to sertraline slowly
       - Escalating doses of prazosin to a target dose of at least 10-15 MG QHS or the dose that attains substantial improvement of nightmares
       - Deprescribe trazadone.
       - Monitor progress with PTSD Check List-5 (PCL-5.)
   - **Complex persistent opioid dependence with protracted withdrawal syndrome:** Explained the mechanisms and treatment. Treatment include the following.
   - Acceptance of the “opioid” induced pain syndrome diagnosis.
     - Treat dependence with sublingual buprenorphine/naloxone 2 MG BID to be escalated based on need for functional improvement. It was explained clearly that this was not to reduce pain but to treat dependence which can result in in improved ability to function. This is to be used to attain maximum function and then patient must retrain to maintain high function with less and less buprenorphine and eventually none. This usually takes 2 years or more.
     - Patient advised to not manage intermittent pain between doses using any pain management. These are expectancy pains and should be managed behaviorally. They high level of pain resolves by itself.
   - **Remitted cannabis and alcohol use disorders:** No further treatment needed.
   - **Polypharmacy:** None of the medications for chronic pain are effective. We will work on weaning off all these medications. Weaning off pain management need is an important part of rehabilitation. Start with gabapentin and then amitriptyline.
   - **Overuse of pain management:** Do not take over the counter pain medications. Slowly wean off regular use of non-pharmacological pain management.

**Follow up:** In one month.
