# Supplementary material for: Conceptualizing and treating the polytrauma clinical triad as a complex chronic pain syndrome
Source: Front Neurol. 2026 Jul 17;17:1752206. doi: 10.3389/fneur.2026.1752206 (PMC13423984; doi:10.3389/fneur.2026.1752206)
Supplement: Supplementary file 2 [file Supplementary_file_2.DOCX]

Appendix 2: Flow charts for the diagnosis of chronic pain syndromes.

Figure 1: Flow chart for establishing a diagnosis of a chronic pain syndrome


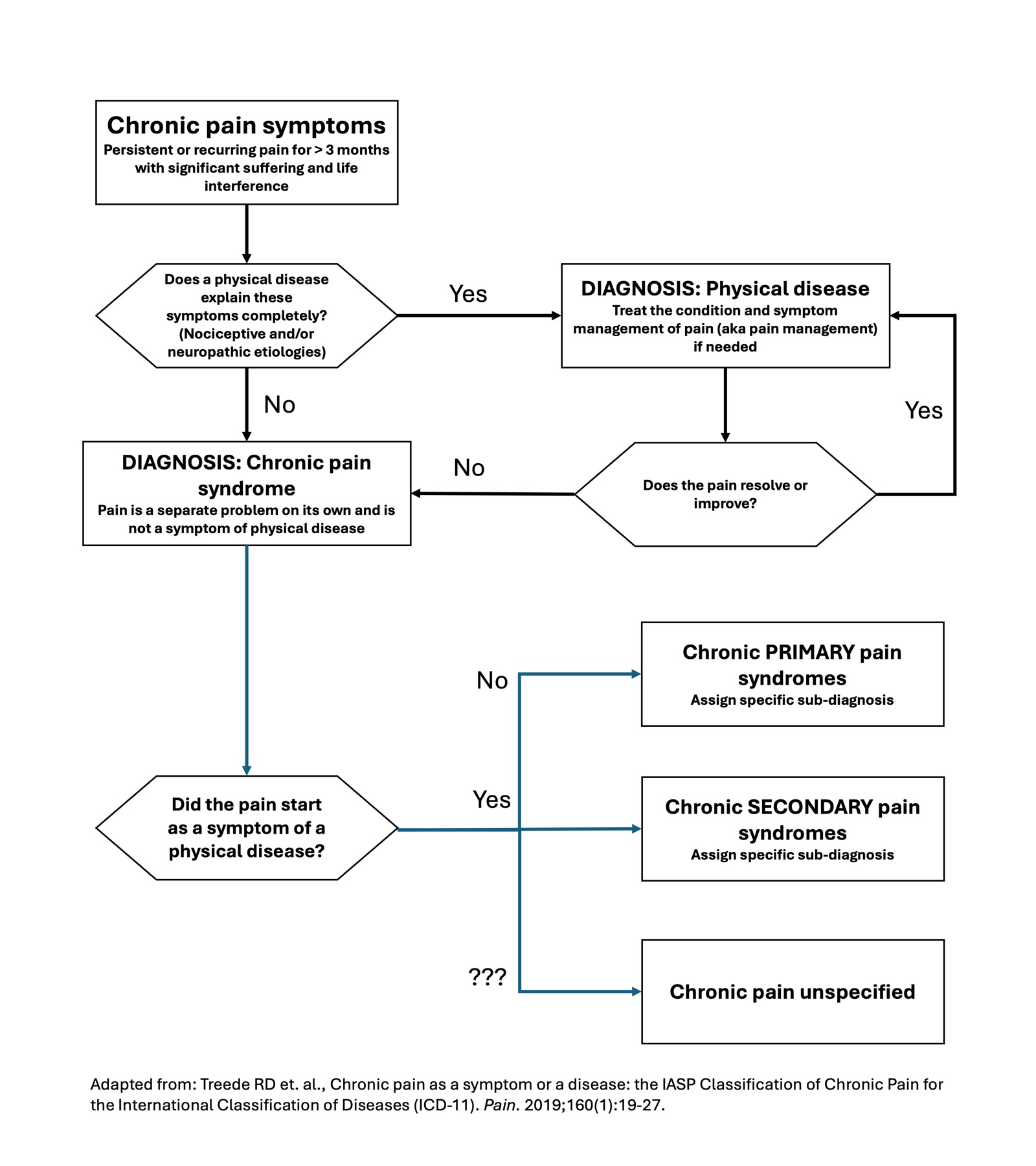


Table 1: Common chronic primary and secondary chronic pain syndromes.

| Chronic Primary Pain syndromes | Chronic Secondary Pain syndromes |
| --- | --- |
| **Chronic widespread pain syndromes** *(fibromyalgia)* | **Chronic cancer related pain**  **Chronic postsurgical or posttraumatic pain**  **Chronic neuropathic pain**  **Chronic secondary headache or orofacial pain**  **Chronic secondary visceral pain**  **Chronic secondary musculoskeletal pain** |
| **Complex regional pain syndrome** *(type I)* |  |
| **Chronic primary headache or orofacial pain**  *Chronic migraine, chronic tension headache, Trigeminal autonomic cephalalgias, chronic temporomandibular disorder pain, chronic burning mouth, chronic orofacial pain* |  |
| **Chronic primary visceral pain**  *Chronic primary chest pain syndrome, chronic primary epigastric pain syndrome, irritable bowel syndrome, chronic primary abdominal pain syndrome, chronic primary bladder pain syndrome, and chronic primary pelvic pain syndrome* |  |
| **Chronic primary musculoskeletal pain** (other than orofacial)  *Chronic primary cervical pain, chronic primary thoracic pain, chronic primary low back pain, and chronic primary limb pain* |  |

Figures and tables developed from: Treede RD, Rief W, Barke A, Aziz Q, Bennett MI, Benoliel R, Cohen M, Evers S, Finnerup NB, First MB, Giamberardino MA, Kaasa S, Korwisi B, Kosek E, Lavand'homme P, Nicholas M, Perrot S, Scholz J, Schug S, Smith BH, Svensson P, Vlaeyen JWS, Wang SJ. Chronic pain as a symptom or a disease: the IASP Classification of Chronic Pain for the International Classification of Diseases (ICD-11). *Pain* 2019;**160**(1)**:** 19-27.
